# Phylogenetic insight into ABCE gene subfamily in plants

**DOI:** 10.3389/fgene.2024.1408665

**Published:** 2024-06-07

**Authors:** Liina Jakobson, Jelena Mõttus, Jaanus Suurväli, Merike Sõmera, Jemilia Tarassova, Lenne Nigul, Olli-Pekka Smolander, Cecilia Sarmiento

**Affiliations:** ^1^ Department of Chemistry and Biotechnology, Tallinn University of Technology, Tallinn, Estonia; ^2^ Department of Biological Sciences, University of Manitoba, Winnipeg, MB, Canada

**Keywords:** ABCE gene subfamily, ABCE, gene evolution, phylogenetics, natural variation

## Abstract

ATP-BINDING CASSETTE SUBFAMILY E MEMBER (ABCE) proteins are one of the most conserved proteins across eukaryotes and archaea. Yeast and most animals possess a single *ABCE* gene encoding the critical translational factor ABCE1. In several plant species, including *Arabidopsis thaliana* and *Oryza sativa*, two or more *ABCE* gene copies have been identified, however information related to plant *ABCE* gene family is still missing. In this study we retrieved *ABCE* gene sequences of 76 plant species from public genome databases and comprehensively analyzed them with the reference to *A. thaliana ABCE2* gene (*AtABCE2*). Using bioinformatic approach we assessed the conservation and phylogeny of plant ABCEs. In addition, we performed haplotype analysis of *AtABCE2* and its paralogue *AtABCE1* using genomic sequences of 1,135 *A. thaliana* ecotypes. Plant ABCE proteins showed overall high sequence conservation, sharing at least 78% of amino acid sequence identity with AtABCE2. We found that over half of the selected species have two to eight *ABCE* genes, suggesting that in plants *ABCE* genes can be classified as a low-copy gene family, rather than a single-copy gene family. The phylogenetic trees of ABCE protein sequences and the corresponding coding sequences demonstrated that *Brassicaceae* and *Poaceae* families have independently undergone lineage-specific split of the ancestral *ABCE* gene. Other plant species have gained *ABCE* gene copies through more recent duplication events. We also noticed that ploidy level but not ancient whole genome duplications experienced by a species impacts *ABCE* gene family size. Deeper analysis of *AtABCE2* and *AtABCE1* from 1,135 *A. thaliana* ecotypes revealed four and 35 non-synonymous SNPs, respectively. The lower natural variation in *AtABCE2* compared to *AtABCE1* is in consistence with its crucial role for plant viability. Overall, while the sequence of the ABCE protein family is highly conserved in the plant kingdom, many plants have evolved to have more than one copy of this essential translational factor.

## 1 Introduction

Members of the ATP-BINDING CASSETTE (ABC) subfamily E (ABCE) belong to the superfamily of ABC proteins, which can be found in all living organisms studied to date and are regarded as highly essential in all eukaryotes. Most ABC proteins function as ATP-dependent membrane transporters. They possess transmembrane domains (TMDs) coupled with nucleotide-binding domains (NBD) otherwise known as ATP-binding cassettes (Andolfo et al., 2015; Navarro-Quiles et al., 2018). ABCE [initially denoted RNASE L INHIBITOR (RLI)] proteins, in contrast, lack TMDs, but still have two NBDs associated with several specific domains and thus are soluble proteins.

In most species the ABCE subfamily is represented by a single member, ABCE1, which is involved in ribosome biogenesis and several stages of translation regulation (Yarunin et al., 2005; Andersen and Leevers, 2007; Barthelme et al., 2011; Mancera-Martínez et al., 2017; Navarro-Quiles et al., 2018). In accordance with its fundamental role, ABCE1 expression has been detected in most tissues and developmental stages of the species studied. In addition, loss-of-function of *ABCE1* genes results in a lethal phenotype in all studied species (Du et al., 2003; Zhao et al., 2004; Maeda et al., 2005; Sarmiento et al., 2006; Kougioumoutzi et al., 2013). ABCE1 has been found to participate in translational initiation and termination, however, its most conserved function is in the process linking these two stages of translation—ribosome recycling (Navarro-Quiles et al., 2018). During that process, ABCE1 splits the ribosome through direct interactions with ribosomal subunits and release factors, either after canonical stop codon-dependent termination or after recognition of stalled and vacant ribosomes. The latter is recognized during mRNA surveillance mechanisms such as no-go decay (NGD), non-stop decay (NSD), and non-functional 18S rRNA decay (18S-NRD) (Graille and Séraphin, 2012). Furthermore, ABCE1 dissociates the 80S-like complex during maturation of ribosomal subunits (Strunk et al., 2012). The role in ribosome biogenesis is supported by the nuclear accumulation of 40S and 60S ribosome subunits in the absence of ABCE1 (Kispal et al., 2005; Yarunin et al., 2005; Andersen and Leevers, 2007). Additionally, it has a key role in RNA silencing in both plants and animals (Sarmiento et al., 2006; Kärblane et al., 2015). Moreover, we have previously shown that human ABCE1 (HsABCE1) is directly or indirectly involved in histone biosynthesis and DNA replication (Toompuu et al., 2016).

The study of ABCE functions in plants has been mostly limited to the model plants *Arabidopsis thaliana*, *Nicotiana benthamiana*, *Nicotiana tabacum* and *Cardamine hirsuta* (Petersen et al., 2004; Sarmiento et al., 2006; Kougioumoutzi et al., 2013; Mõttus et al., 2021; Navarro-Quiles et al., 2022). In *A. thaliana* there are two genes encoding for paralogous ABCE proteins (AtABCE1 and AtABCE2, also referred to as AtRLI1 and AtRLI2, respectively), which share 80.8% identity (Mõttus et al., 2021; Navarro-Quiles et al., 2022). AtABCE2 is orthologous to HsABCE1 and is ubiquitously expressed in all plant organs (Sarmiento et al., 2006). Recently, AtABCE2 was found to interact with ribosomal proteins and translational factors, confirming its conserved ancestral function in translation that is coupled to general growth and vascular development, likely indirectly via auxin metabolism (Navarro-Quiles et al., 2022). Furthermore, through regulation of translation AtABCE2 is involved in the development of gametophyte and embryo (Yu, et al., 2023). In addition, AtABCE2 has been shown to suppress GFP transgene RNA silencing in heterologous system at the local and at the systemic levels by reducing accumulation of siRNAs (Sarmiento et al., 2006; Kärblane et al., 2015). Mutational analysis of AtABCE2 revealed that the structural requirements for RNA silencing suppression are similar to those needed for ribosome recycling in archaea (Mõttus et al., 2021). This indicates that AtABCE2 might suppress RNA silencing via supporting translation-associated RNA degradation mechanisms. The role of AtABCE1 in *A. thaliana*, which is expressed almost exclusively in generative organs (Navarro-Quiles et al., 2022; Yu et al., 2023), is yet to be studied.

Silencing of *ABCE* orthologues *(RLIh)* in *N. tabacum* resulted in a single viable transgenic plant exhibiting severe morphological alterations, supporting the important role of ABCE proteins at the whole-organism level. At that time, it remained unclear how many *RLIh* genes there are in tobacco species (Petersen et al., 2004). In *C. hirsuta,* a close relative of *A. thaliana* that has composite leaves, there is only one *ABCE* gene in the genome, named SIMPLE LEAF3 (SIL3, or ChRLI2) (Kougioumoutzi et al., 2013). Hypomorphic mutation Pro177Leu in the NBD1 domain of ChRLI2 affects the determination of leaf shape and regulation of auxin homeostasis (Kougioumoutzi et al., 2013). Interestingly, the expression of *ChRLI2* was not ubiquitous as in *A. thaliana*, but instead it was shown to be expressed in meristematic and vascular tissues of young developing leaves and in leaflet initiation sites (Kougioumoutzi et al., 2013).

It is commonly claimed that most eukaryotes only have one *ABCE* gene (Dermauw and Van Leeuwen, 2014). Exceptions to this have been detected in plants such as thale cress, rice, maize, potato and tomato, but also in animals such as catfish, cod and mosquitoes (Braz et al., 2004; Garcia et al., 2004; Verrier et al., 2008; Liu et al., 2013; Pang et al., 2013; Andolfo et al., 2015; Lu et al., 2016). Although some plant species have more than one *ABCE* gene, it is still the smallest and most conserved of all ABC subfamilies (Andolfo et al., 2015).

In this study we aimed to characterize the phylogenetic evolution of *ABCE* genes in plants in order to shed light on the possible functional diversification within ABCE protein family. Here we present the results of an extensive bioinformatics analysis of publicly available sequences for plant *ABCE* genes and corresponding proteins, together with haplotype analysis of *A. thaliana ABCE*s.

## 2 Methods

### 2.1 Construction of the phylogenetic diagram

The phylogenetic diagram of the studied plants species together with the bar chart of *ABCE* gene number was created based on NCBI taxonomy with phyloT and visualized with iTOL (Letunic and Bork, 2007; 2019; phylot.biobyte.de).

### 2.2 Genome and proteome data acquisition

ABCE sequence data for 55 species was downloaded from the online resource Phytozome portal https://phytozome.jgi.doe.gov/ (Goodstein et al., 2012). ABCE sequence data for additional 18 species was downloaded from Ensembl Plants (Howe et al., 2020). The genome data for *C. hirsuta* was accessed at http://bioinfo.mpipz.mpg.de/blast/ (Gan et al., 2016). Genome data for *N. tabacum* and *N. benthamiana* was downloaded from Sol Genomics Network http://solgenomics.net (Edwards et al., 2017; Kourelis et al., 2019). We downloaded genomic, CDS and translated amino acid sequences for each plant *ABCE* gene used in the study.

The length of amino acid sequences was calculated with SeqinR package (version 3.6.1) in R 4.0.2 (Charif and Lobry, 2007). In order to calculate their similarities to AtABCE2, all 152 sequences were aligned using the online interface of MUSCLE with default Pearson/FASTA parameters provided by the European Bioinformatics Institute (EBI) (Madeira et al., 2019). Thereafter the percent identity scores were calculated with MUSCLE algorithm for aligned sequences in R 4.0.2 package Bio3D version 2.4-1 (Edgar, 2004; Grant et al., 2021).

Next, sequences aligned to AtABCE2 were inspected for the general protein structure, that is the presence and correct order of the domains, including iron-sulphur (FeS) cluster domain, NBD1, NBD2 and bipartite Hinge domain (Supplementary Figure S1). Sequences lacking critical motifs within these domains (Karcher et al., 2005; Barthelme et al., 2007; Nürenberg and Tampé, 2013; Nürenberg-Goloub et al., 2020) were filtered out from the analysis.

### 2.3 Evolutionary analysis by maximum likelihood method

Amino acid sequences were aligned with default parameters of the MUSCLE algorithm, as implemented in the MEGA software package (version 11.0.13) (Tamura et al., 2021). CDS sequences were aligned with the default parameters of MAFFT (version 7.4.9.0) (Katoh and Standley, 2013). As the alignments contained gaps, sites with no data for more than 10% of the sequences were removed with trimAl (version 1.4. rev22) (Capella-Gutiérrez et al., 2009), resulting in an amino acid alignment of 603 positions and a CDS alignment of 1,810 positions.

Phylogenetic trees of ABCE full length amino acid sequences and the corresponding CDS sequences were constructed by IQ-TREE (version 2.0.7) (Minh et al., 2020) with the Maximum Likelihood method and 10,000 rapid bootstrap replicates. Initial tests suggested that the best models to use would be JTTDCMut for amino acids and TIM2e for CDS sequences, both with five degrees of FreeRate heterogeneity (Soubrier et al., 2012). Tree calculation was performed 20 times independently for both CDS and amino acid input, with random seed values ranging from 1 to 20. TreeGraph (version 2.15.0–887) (Stöver and Müller, 2010) was used to root all trees on *Chlorophyta* and collapse any branches supported by bootstrap values of 50% or less. The trees were then all added to a single file in the order of their log likelihoods (highest to lowest) and annotated in FigTree (version 1.4.3; http://tree.bio.ed.ac.uk/software/figtree/). Final adjustments (fine-tuning the color scheme) were done in Adobe Illustrator.

Unrooted tree supporting suitability of *Chlorophyta* as an outgroup as well as separate trees based on the amino acid sequences of ABCE domains were constructed by MEGA software. Phylogenetic relationships were inferred using the Maximum Likelihood method and JTT matrix-based model (Jones et al., 1992), selected for each data set based on the lowest BIC scores (Bayesian Information Criterion). A discrete gamma distribution was used to model evolutionary rate differences among sites. Bootstrap analysis was performed with 500 replicates. The trees with the highest log likelihood were published for each analysis.

### 2.4 Modelling the drivers of ABCE copy number

Two different approaches were used to test the impact of assembly size, ploidy level, and the number of known ancient whole genome duplications on ABCE copy numbers. A simple linear regression was performed with the lm function available in base R (version 4.3.2) (R Core Team, 2023). In an alternative approach, the R package taxize (version 0.9.100) (Pinheiro et al., 2023) was first used to extract phylogenetic relationships of all species involved from the NCBI databases. Next, the function gls (generalized least squares) from R package nlme (version 3.1–164) (Pinheiro and Bates, 2000; Pinheiro et al., 2023) was used to create additional regression models with phylogenetic signal included as a random effect. Ggpredict from the R package ggeffects (version 1.3.4) (Lüdecke, 2018) was used to obtain prediction intervals for the models. The results were plotted with the packages ggplot2 (version 3.4.2) (Wickham, 2016) and patchwork (version 1.2.0) (Pedersen, 2024), then adjusted in Adobe Illustrator.

### 2.5 Data acquisition for 1135 Arabidopsis ecotypes

Data (SNPs and indels) available for 1135 *A. thaliana* strains was downloaded from the 1,001 Genomes Project depository (Weigel and Mott, 2009).

### 2.6 Reconfirming the haplotypes of Arabidopsis ecotypes

Seeds of the selected 21 Arabidopsis ecotypes were acquired from the Nottingham Arabidopsis Stock Centre (NASC). Both *AtABCE1* and *AtABCE2* full coding sequences were PCR-amplified and sequenced by Sanger sequencing for ecotypes Can-0, Ei-2, IP-Car-1, Kia1 and Pra-6. For the other 15 ecotypes only *AtABCE1* full coding sequences was sequenced. Primer pairs used for the PCRs are shown in the Supplementary Table S1. For all the PCR reactions touchdown PCR method with the following conditions was used: 95°C for 15 min; 13 cycles of at 95°C for 15 s, at the gradually decreasing temperature from 60°C to 54°C (the temperature drops by 0.5°C per cycle) for 30 s, at 72°C for 70 s; 15 cycles of 95°C 15 s, 54°C 30 s, 72°C 70 s and the final extension at 72°C for 10 min. Amplified DNA fragments were purified from the agarose gel using GeneJET Gel Extraction Kit (Thermo Scientific) according to the manufacturer’s instructions. Thereafter the purified DNA fragments were Sanger sequenced and aligned respectively to *AtABCE1* or *AtABCE2*. The final results were based on the sequencing of at least two plants for each ecotype. Columbia (Col-0) ecotype was used as a reference.

### 2.7 Generating haplotype map with PopART

For the haplotype analysis, first an alignment file with CDS sequences was created in Nexus format. Thereafter the multiple sequence alignment was analyzed with PopART version 1.7 (Population Analysis with Reticulate Trees) (Leigh and Bryant, 2015). The network was constructed with Median Joining Network algorithm (epsilon = 0).

## 3 Results

### 3.1 Variability of plant *ABCE* genes

**FIGURE 1:**
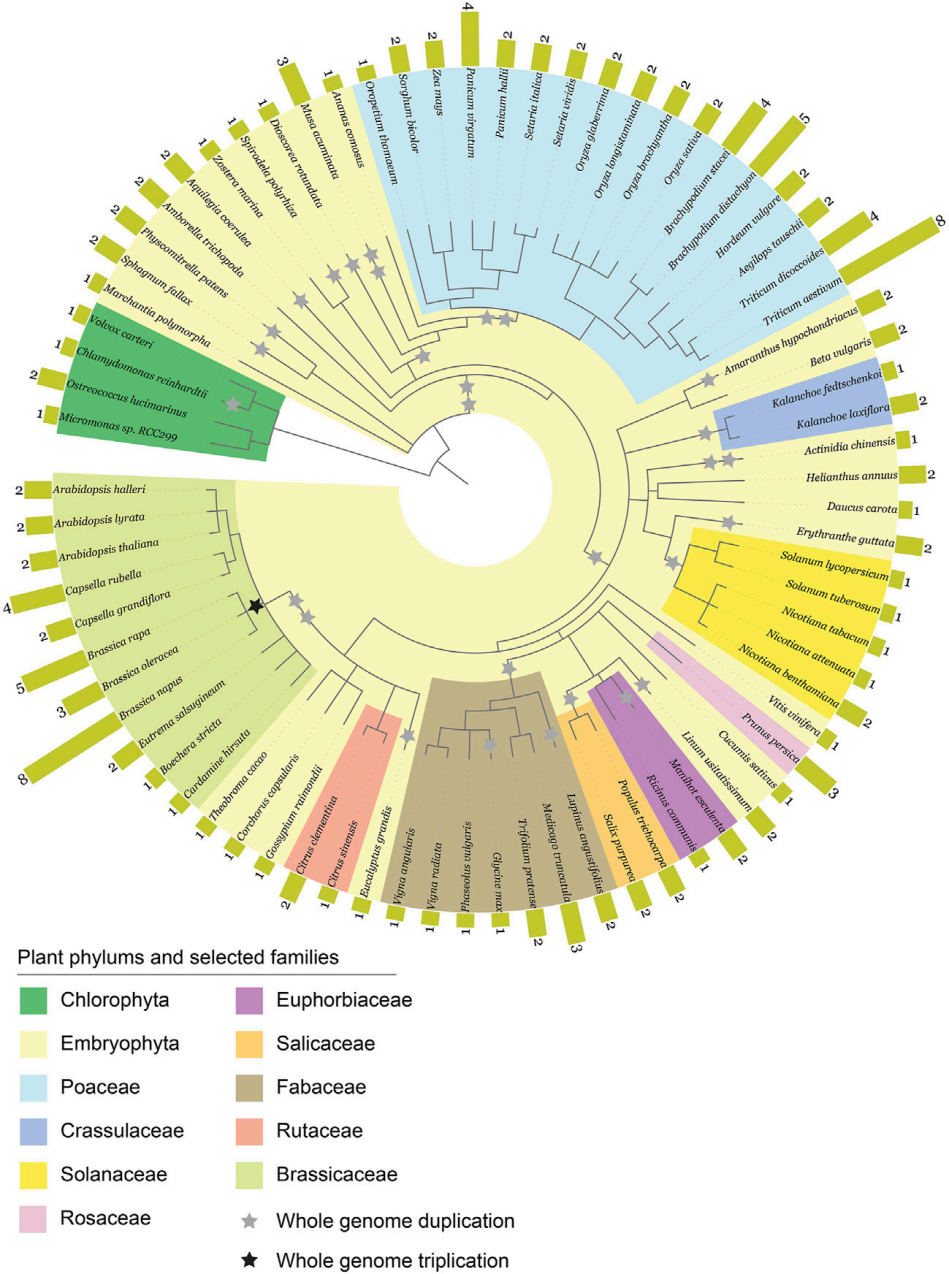
Phylogenetic diagram of 152 *ABCE* genes in plants. The data was compiled from 76 plant species. Whole genome duplications (WGDs) and triplications (WGT) are marked as grey and black stars, respectively.

**FIGURE 2:**
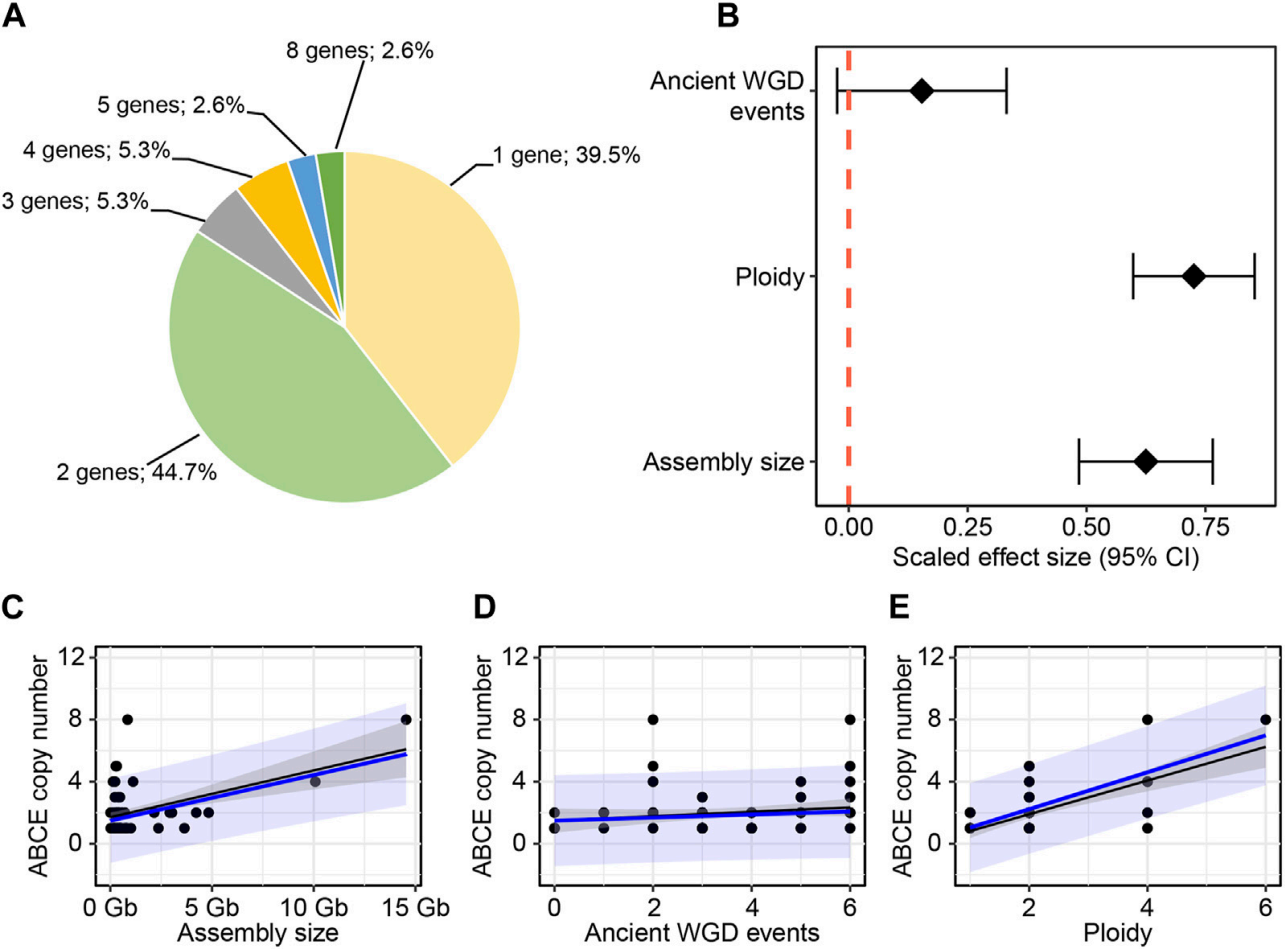
Relationship between *ABCE* copy number and genomic parameters. **(A)** *ABCE* gene copy number among the studied 76 plant species. **(B)** Both ploidy level and the size of the genome assembly have a positive effect on the number of *ABCE* genes. The number of ancient whole genome duplications has no clear effect. **(C–E)** Correlation between *ABCE* copy number and different predictor variables. Black: linear regression, with 95% confidence intervals. Blue: phylogenetic generalized least squared regression, with 95% prediction intervals.

To gain insight into the diversity of *ABCE* genes in plants, we compiled a selection of *ABCE* genes from 76 different plant species available in public databases. The selection criteria for including in further analysis was the presence of all known essential structural elements of ABCE proteins (Karcher et al., 2005; Barthelme et al., 2007; Nürenberg and Tampé, 2013; Nürenberg-Goloub et al., 2020). Truncated or aberrant sequences were discarded from further analysis. Altogether 152 plant *ABCE* genes were included in the study. We found that the amino acid sequence identity among the 152 proteins was 78% or higher when compared to *A. thaliana* AtABCE2. The selected species represented a wide range of plant groups, including unicellular algae such as *Chlamydomonas reinhardtii* and *Micromonas* sp. *RCC299*, monocots such as *Zea mays* and *Triticum aestivum*, *Solanum* species such as *Solanum tuberosum* and *N. benthamiana*, and *Brassicaceae* such as *Brassica napus* and *A. thaliana* (Figure 1; Supplementary Table S2). Our analysis revealed that plant species from the phylum *Chlorophyta* (green algae) usually possess only a single *ABCE* gene, except for *Ostreococcus lucimarinus*, which has two genes. In contrast, most of the analyzed species in the *Poaceae* family have at least two *ABCE* genes, and some have as many as eight genes in their genome, as is the case for *T. aestivum*. Another group of plant species with an above-average number of *ABCE* genes is the *Brassicaceae* family. For example, *B. napus* has eight genes, *B. rapa* five genes, and *Capsella rubella* four genes. On the other hand, among *Brassicaceae*, *C. hirsuta* and *Boechera stricta* have only a single *ABCE* gene. Despite clustering of multi-gene-species in the *Poaceae* and *Brassicaceae* families, there was no visual segmentation between the number of *ABCE* genes and phylogenetic origin in other plant families (Figure 1). Interestingly, only 30 species out of 76 (39.5%) had a single *ABCE* gene. Similarly, there were 34 species (44.7%) possessing two *ABCE* genes (Figure 2A). This shows that despite some species having only single functional *ABCE* gene (containing full set of critical structural elements) *ABCE* genes in plants can be classified as a low-copy gene family instead of single-copy gene family.

Our next goal was to explore how genomic parameters contribute to *ABCE* gene copy number in plants. We tested the effect of ploidy level, genome size and WGD events during evolution on the *ABCE* gene family size of a species. WGD data was based on the data of 53 plant species published by the One Thousand Plant Transcriptomes Initiative (Leebens-Mack et al., 2019). The observation of WGT data in the common ancestor of *Brassica* species was based on the study of Wang and coworkers (Wang et al., 2011). Regression analysis showed positive effect of ploidy level and genome size on *ABCE* gene copy number. Among selected parameters a degree of ploidy is likely the most suitable as a prediction factor, although there were examples of tetraploid species with a single *ABCE* gene (e.g., *Nicotiana tabacum*) and diploids with five *ABCE* genes (e.g., *Brachypodium distachyon*, *B. rapa*) (Figures 2B,C,E; Supplementary Table S2). In contrast, there was no clear correlation between ancient WGD events experienced by a species and *ABCE* gene family size (Figures 2B,D; Supplementary Table S2). Interestingly, there are examples of species, which have encountered at least five WGDs in their evolutionary history, but still possess only a single *ABCE* gene, for example, *Actinidia chinensis* and *Glycine max* (Figure 1; Supplementary Table S3).

### 3.2 Plant *ABCE*s are highly conserved

**FIGURE 3:**
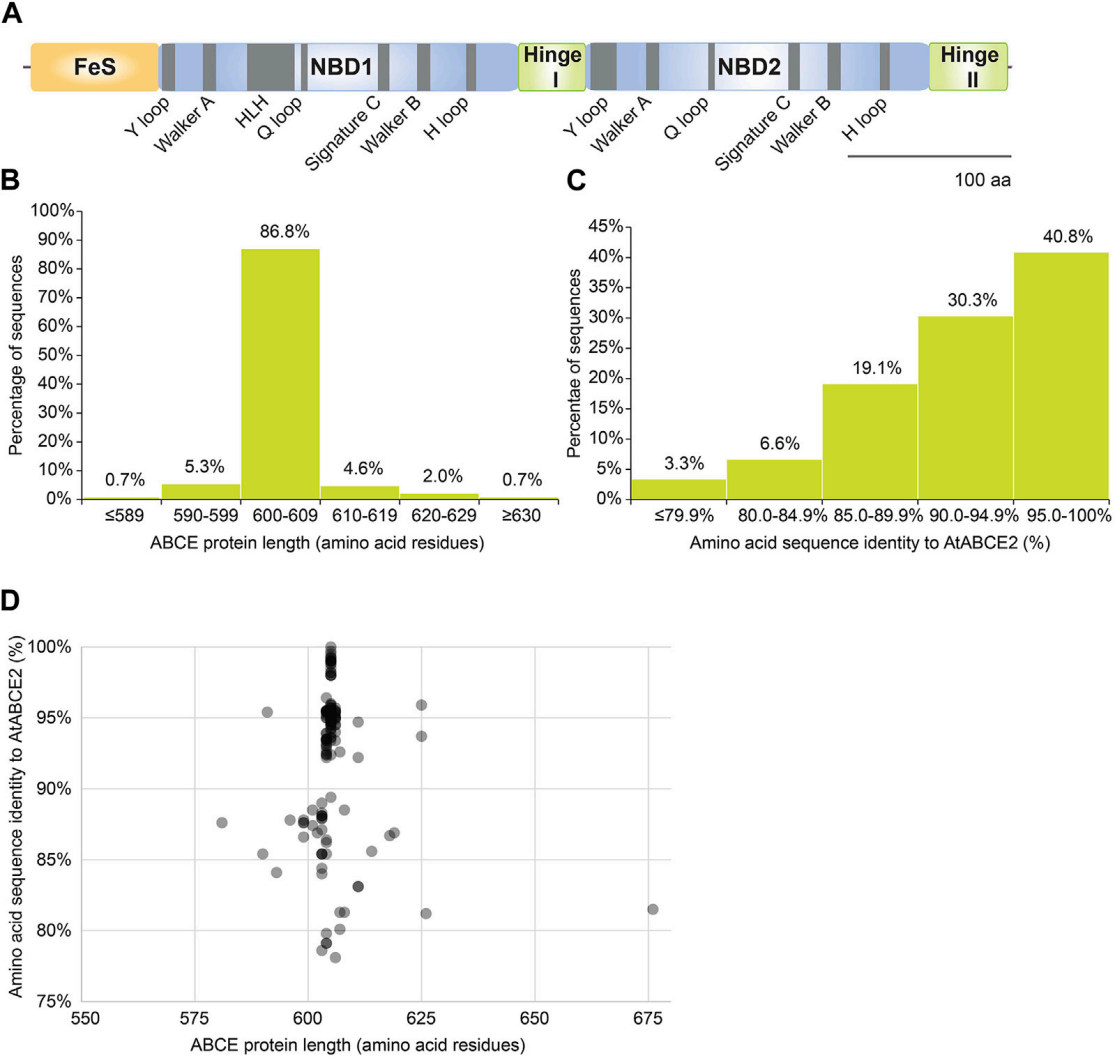
Amino acid sequences of plant ABCEs reveal high level of conservation. **(A)** Linear protein model of AtABCE2. Grey regions depict highly conserved motifs within AtABCE2. FeS—iron-sulphur cluster domain, NBD1—nucleotide-binding domain 1, NBD2—nucleotide-binding domain 2. **(B)** Histogram of protein sequence lengths of the studied 152 plant ABCEs. **(C)** Histogram of amino acid sequence identities of the studied 152 plant ABCEs, based on MUSCLE alignment. **(D)** The correlation between amino acid sequence identity and protein sequence length of the studied 152 plant ABCEs.

ABCE proteins are composed of four domains: NBD1 and NBD2 forming the ATPase core, bipartite hinge domain that is tightly engaged in twin-NBD cassette arrangement and a unique N-terminal FeS cluster domain (Figure 3A). Additionally, ABCEs embody a helix–loop–helix (HLH) motif in NBD1 that distinguishes it from otherwise superimposable NBD2 (Karcher et al., 2005).

As many as 86.8% out of the 152 analyzed gene sequences encode ABCE of canonical protein length (600—609 amino acids) (Figure 3B). Along with exceptional conservation within functionally critical motifs, all analyzed ABCE sequences are highly similar to AtABCE2 sharing at least 78% of amino acid sequence identity (Figure 3C; Supplementary Table S4).

We also plotted amino acid sequence length to amino acid sequence identity for the studied 152 ABCE proteins. There was a clear clustering of proteins with the length of 605 amino acids (Figure 3D). Proteins with lower sequence identity did not cluster by protein length (Figure 3D). Interestingly, proteins with more than 90% identity to AtABCE2 could be as short as 591 amino acids and as long as 625 amino acids long (Figure 3D). Hence, despite some variance in amino acid sequence length and sequence identity to AtABCE2, the selection of amino acid sequences analyzed here is uniform and represents well the plant *ABCE* genes.

### 3.3 Phylogeny of plant *ABCE*s

**FIGURE 4:**
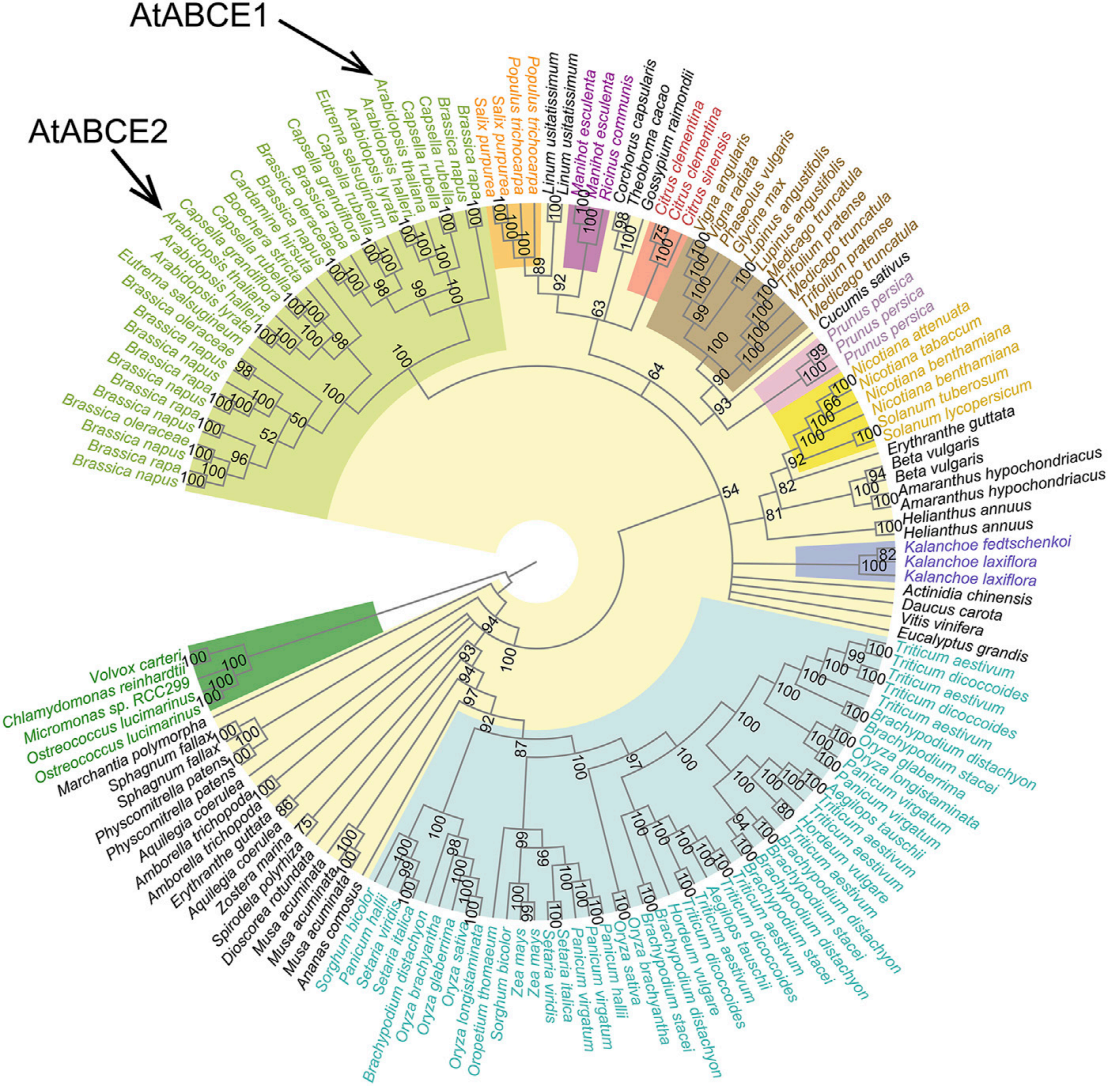
Cladogram of the 152 plant *ABCE* full-length CDS sequences. There were a total of 1,810 positions in the final dataset. The tree was constructed using the Maximum Likelihood method, TIM2e model with five categories of FreeRate heterogeneity and 10,000 rapid bootstrap replicates. All branches supported by bootstrap values of less than 50% were collapsed. Color-coding refers to affiliation with larger plant phyla or families as indicated in Figure 1. The tree is rooted on the phylum *Chlorophyta*.

To understand how ABCE proteins have evolved in the green plant lineage, we constructed 20 Maximum Likelihood (ML) trees of 152 full-length ABCE protein sequences and the corresponding coding DNA (CDS) sequences from 76 species (Figure 4; Supplementary Figures S2–S4). *Chlorophyta* (green algae), the earliest lineage to have split off from the rest of the green plants, was used as an outgroup for rooting the trees. In an unrooted tree the representatives of *Chlorophyta* formed a separate cluster with high bootstrap support (Supplementary Figure S5).

The CDS tree (Figure 4; Supplementary Figure S4) proved much more informative than the amino acid tree (Supplementary Figures S2, S3). Most internal branches in the amino acid tree are poorly supported by bootstrap values, likely resulting from the lack of phylogenetic signal in the highly conserved sequences. The CDS tree is more congruent with the known species tree (Figure 1). However, both show that the sequences cluster according to major taxonomic groupings, usually with high bootstrap support. For instance, all ABCEs from *Fabaceae* (legumes) form a single cluster, as is the case for *Solanaceae* (nightshades), *Poaceae* (grasses), *Brassicaceae* (mustard and cabbage family, including the thale cress *A. thaliana*), and others. These groupings appear older than any duplication events present in the ABCE family. For example, the *AtABCE1* and *AtABCE2* genes of *A*. *thaliana* are the result of a duplication that happened in *Brassicaceae*, whereas the multiple copies seen in wheat and other members of *Poaceae* result from different duplication events. Further examination of the data revealed that all cases with more than one ABCE sequence in a given species can be broadly divided in two. Often the closest neighbor for one of the sequences was a different one from the same species, which is most likely reflective of recent duplication events. However, in other cases much older duplications were found, with two or more gene copies evolving independently across different species of the same plant family or order. This is well known for *ABCE* genes in *Brassicaceae* (Navarro-Quiles et al., 2022), and our analysis confirms that many of their family members including *A. thaliana* encode distinct ABCE1 and ABCE2 (Figure 4). In support of the notion that AtABCE2 preserves the ancestral function (Navarro-Quiles et al., 2022), we show that ABCE2 sequences have fewer mutations and shorter branch lengths compared to ABCE1s in the phylogenetic tree (Supplementary Figure S4). Notably, all *Brassicaceae* species have at least one ABCE2 protein while ABCE1 can be missing.

Similarly to the ABCE1 of *Brassicaceae*, *Poaceae* (grasses) also include one set of ABCEs that have acquired more mutations than the others. It is present in all analyzed species of rice (*Oryza*), in foxtails (*Setaria*), in sorghum and in the common grasses *Panicum hallii* and *Brachypodium distachyon*. All of those also have at least one slower evolving copy. Wheat and related species (*Triticum*) do not have a direct homolog of the fast-evolving ABCE copy, but they have multiple ABCEs regardless. In any case, the fast-evolving ABCE of *Poaceae* is not the direct homologue of the ABCE1s in *Brassicaceae*, and it is much closer to other ABCEs in *Poaceae* instead. Thus, both *Poaceae* and *Brassicaceae* have at least two distinct ABCE lineages that appeared in the ancestors of the respective families. In both cases there is evidence of rapid accumulation of mutations in one of the genes (long branches in the phylogeny), likely reflective of neo- or subfunctionalization. In amino acid trees (but not CDS trees) those two unrelated groups stemming from long branches are typically grouped together, which is likely due to long branch attraction. In most such cases, one of the three genes from the plum *Prunus persica* also tends to group together with those two sets of sequences (as seen from supplementary trees presented in the associated GitHub repository: https://github.com/jsuurvali/abce152). As expected, the plum gene originates from a longer branch than the other two genes in that species.

In contrast to *Brassicaceae* and *Poaceae*, based on our dataset no such ancestral subtype separation was found in other plant clades. For example, the model species *N. benthamiana* has two ABCEs, but some *Solanaceae* have only one, and all ABCEs in *Solanaceae* are closer to each other than to any of the ones in *Brassicaceae* or *Poaceae* (Figure 4; Supplementary Figure S4). However, in CDS tree the placement of some groups in relation to each other could not be resolved from ABCE sequences alone and was both incongruent with their known positions in the Tree of Life and poorly supported by bootstrap analyses. This is the case for the relationship between *Brassicaceae* and other representatives of Rosids (including *Citrus* sp.). The placement of *ABCE* sequences from *Amborella trichopoda*, a single extant member of a sister lineage to all other angiosperms, did not match the species tree as well (Figure 4; Supplementary Figure S4). However, the effect of those artifacts was reduced by collapsing poorly supported branches in the tree, and showing then clearly that the exact relationship between *Brassicaceae* and other eudicots, or *A. trichopoda* and other angiosperms cannot be fully resolved based on the data. In the current version, those branches and groupings appear as part of polytomies in a multifurcating tree.

In addition, we separately analyzed the three main domains of ABCE proteins, FeS cluster domain, NBD1, and NBD2, by realigning the corresponding amino acid sequences and constructing a ML-tree for each (Supplementary Figures S6–S8). The topology of the resulting trees was different for each domain, but none of those were well supported by bootstrap analysis.

### 3.4 Natural variation of Arabidopsis *AtABCE1* and *AtABCE2* genes

**FIGURE 5:**
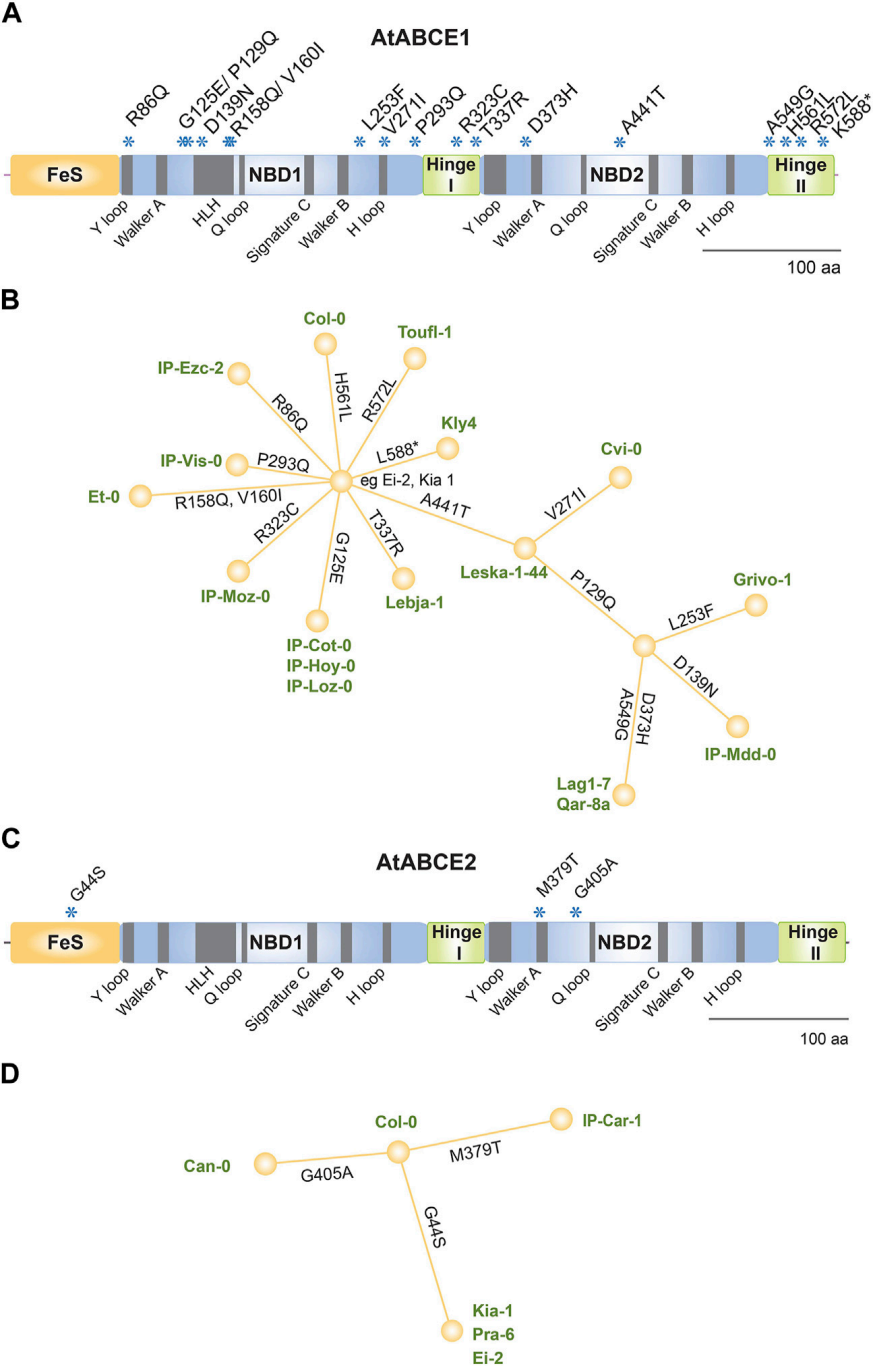
Analysis of non-synonymous SNPs in *AtABCE1* and *AtABCE2*. **(A)** Linear protein model of AtABCE1. Asterisks depict amino acid substitutions due to SNPs verified in different ecotypes. **(B)** Haplotype map of AtABCE1 detected among 22 Arabidopsis ecotypes. Branch length represents the number of mutations between sequences. For pairs of haplotypes whose distances on the tree are longer than the distances between the sequences, edges are added to shorten the distance. **(C)** Linear protein model of AtABCE2. Asterisks depict amino acid substitutions due to SNPs verified in different ecotypes. **(D)** Haplotype map of AtABCE2 detected among six Arabidopsis ecotypes. Branch length represents the number of mutations between sequences. For pairs of haplotypes whose distances on the tree are longer than the distances between the sequences, edges are added to shorten the distance.

Natural variation among *A. thaliana* ecotypes has been well documented by the 1,001 Genomes Project (Weigel and Mott, 2009). We analyzed the *ABCE* gene sequences of all 1135 *A. thaliana* ecotypes reported in that project and found 35 and four non-synonymous SNPs in *AtABCE1* and *AtABCE2*, respectively (Supplementary Table S5). Only four reported non-synonymous SNPs in *AtABCE2* indicate a low degree of natural variation, which is consistent with its fundamental, conserved role in growth and development. On the other hand, 35 non-synonymous SNPs annotated for *AtABCE1* show relatively higher natural variation. This finding is in agreement with the results from the transspecies phylogenetic analysis of *Brassicaceae* ABCE1 and ABCE2. From the previously reported SNPs in AtABCE1, we selected 18 that cause amino acid substitutions at conserved and important positions or that were present in combination with other SNPs of interest. Therefore, 21 ecotypes were included in the further study and resequencing, together with Col-0 (Table 1; Supplementary Table S5). Two SNPs causing amino acid substitution could not be detected by resequencing (Pro399Thr in Grivo-1 and Gly182Ser in IP-Cot-0). Instead, one SNP previously undocumented in the 1,001 Genomes Project database (Leu253Phe in Grivo-1) was identified. Figure 5A shows the positions of the amino acid substitutions caused by the 17 SNPs sequenced in the *AtABCE1* gene. In our resequencing analysis, the most frequent SNPs in *AtABCE1* caused the changes His561Leu and Ala441Thr (Table 1; Supplementary Table S5). Noteworthy, histidine at the position 561 seems to be characteristic to Col-0, as all the other analyzed ecotypes had leucine at this position. Next, we performed haplotype analysis with CDS sequences on the PopART platform and found that the most conserved sequence of *AtABCE1* is most probably the one identical to Ei-2, Kia1, Pra-6, IP-Car-1 and Can-0. Eight SNPs out of 17 appear as single SNPs in the *AtABCE1* of Kly4, Toufl-1, Col-0, IP-Ezc-2, IP-Vis-0, IP-Moz-0, IP-Hoy-0, IP-Loz-0, IP-Cot-0 and Lebja-1. Interestingly, a substitution of Ala441Thr can appear both as the consequence of a single SNP in Leska-1-44 and together with other SNPs such as in Cvi-0 or Qar-8a (Figure 5B). Some amino acid changes, like Pro129Gln, Ala549Gly and His561Leu, are always grouped (Table 1). Pro129Gln appears only together with at least two other SNPs, e.g., in Grivo-1 or Qar-8a (Figure 5B).

**TABLE 1:** Non-synonymous SNPs found in *AtABCE1* and *AtABCE2* among 21 *A. thaliana* ecotypes. All SNPs were verified by Sanger sequencing and whole-genome sequencing published in the 1,001 Genomes project (Weigel and Mott, 2009; Cao et al., 2011). SNP locations were numbered according to the position in the cDNA sequence starting from ATG. Change in amino acid sequence corresponding to the SNP is presented. Orange color depicts SNPs that are not present in 1,001 Genomes Project data but verified by Sanger sequencing within this study. Green color shows SNPs positioned in conserved arginine residues.

No	Ecotype	SNPs in *AtABCE1* cDNA	Amino acid change in AtABCE1	SNPs in *AtABCE2* cDNA	Amino acid change in AtABCE2	Origin
1	**IP-Ezc-2**	257G>A	Arg86Gln	None	None	Spain
		1682A>T	His561Leu			
2	**IP-Cot-0**	374G>A	Gly125Glu	None	None	Spain
		1682A>T	His561Leu			
3	**IP-Hoy-0**	374G>A	Gly125Glu	None	None	Spain
		1682A>T	His561Leu			
4	**IP-Loz-0**	374G>A	Gly125Glu	None	None	Spain
		1682A>T	His561Leu			
5	**IP-Vis-0**	878C>A	Pro293Gln	None	None	Spain
		1682A>T	His561Leu			
6	**IP-Moz-0**	967C>T	Arg323Cys	None	None	Spain
		1682A>T	His561Leu			
7	**Lebja-1**	1010C>G	Thr337Arg	None	None	Russia
		1682A>T	His561Leu			
8	**Leska-1-44**	1321G>A	Ala441Thr	None	None	Bulgaria
		1682A>T	His561Leu			
9	**Toufl-1**	1682A>T	His561Leu	None	None	Morocco
		1715G>T	Arg572Leu			
10	**Kly4**	1682A>T	His561Leu	None	None	Russia
		1762A>T	Lys588STOP			
11	**Et-0**	473G>A	Arg158Gln	None	None	France
		478G>A	Val160Ile			
		1682A>T	His561Leu			
12	**Cvi-0**	811G>A	Val271Ile	None	None	Cape Verde
		1321G>A	Ala441Thr			
		1682A>T	His561Leu			
13	**IP-Mdd-0**	386C>A	Pro129Gln	None	None	Spain
		415G>A	Asp139Asn			
		1321G>A	Ala441Thr			
		1682A>T	His561Leu			
14	**Grivo-1**	386C>A	Pro129Gln	None	None	Bulgaria
		757C>T	Leu253Phe			
		1321G>A	Ala441Thr			
		1682A>T	His561Leu			
15	**Lag1-7**	386C>A	Pro129Gln	None	None	Georgia
		1117G>C	Asp373His			
		1321G>A	Ala441Thr			
		1646C>G	Ala549Gly			
		1682A>T	His561Leu			
15	**Qar-8a**	386C>A	Pro129Gln	None	None	Lebanon
		1117G>C	Asp373His			
		1321G>A	Ala441Thr			
		1646C>G	Ala549Gly			
		1682A>T	His561Leu			
17	**Ei-2**	1682A>T	His561Leu	130G>A	Gly44Ser	Germany
18	**Kia 1**	1682A>T	His561Leu	130G>A	Gly44Ser	Sweden
19	**Pra-6**	1682A>T	His561Leu	130G>A	Gly44Ser	Spain
20	**IP-Car-1**	1682A>T	His561Leu	1136T>C	Met379Thr	Spain
21	**Can-0**	1682A>T	His561Leu	1214G>C	Gly405Ala	Spain

From the previously reported four non-synonymous SNPs in *AtABCE2*, we were able to reconfirm three SNPs causing amino acid changes (Gly44Ser, Met379Thr and Gly405Ala), which were located in FeS cluster domain and NBD2 at non-conserved positions (Figure 5C; Supplementary Table S5). Asp189Glu in AtABCE2 was not possible to reverify due to unavailable seed material. The verified SNPs were present in five different ecotypes (Table 1). The haplotype map of *AtABCE2* SNPs shows that Col-0 has the most conserved sequence and the three different SNPs root from it (Figure 5D).

In this study we could not find any correlation between the presence of non-synonymous SNPs in *AtABCE* genes and the geographical origin of the ecotype (Table 1). Visual rosette phenotype of the studied ecotypes matched with characterization available in the public databases (Supplementary Figure S9).

In the case of 18 out of 21 ecotypes, all SNPs were confirmed as reported earlier. For three ecotypes only part of the SNPs was validated: Leska-1-44 did not exhibit Pro129Gln, IP-Cot-0 did not exhibit Gly182Ser and Grivo-1 did not exhibit Pro399Thr amino acid changes in AtABCE1 (Supplementary Table S5; Table 1). More interestingly, we verified Gly125Glu in IP-Cot-0 and Pro129Gln, Leu253Phe, Ala441Thr and His561Leu in Grivo-1. Leu253Phe had not been annotated in any *A. thaliana* ecotype in the 1,001 Genomes Project database (Table 1).

Surprisingly, we noticed some SNPs affecting highly conserved amino acid residues in AtABCE1. These include arginine residues from R cluster of Hinge domains (Arg323Cys and Arg572Leu of IP-Moz-0 and Toufl-1, respectively), and Arg86Gln of IP-Ezc-2 ecotype that locates to the Y-loop I (Figure 5A; Table 1). According to the 1,001 Genomes Project database the latter SNP is present in 19 *A. thaliana* ecotypes (Supplementary Table S4).

Although, the length of ABCE proteins in *A. thaliana* ecotypes is very conserved, in a single ecotype, namely, Kly-4, we found a SNP in *AtABCE1* causing premature stop codon that makes the protein 14 amino acid residues shorter. Despite this deletion, the cluster of arginine residues remains intact in Hinge II subdomain (Figures 5A,B; Supplementary Figure S1).

## 4 Discussion

The availability of high-quality plant genome sequences is growing day by day, which creates a completely new and underexploited repository. It has been recognized that the plant genome evolution has been very complex, including polyploidy, periods of rapid speciation and extinction (Leebens-Mack et al., 2019; Wang et al., 2021; Qiao et al., 2022). Interestingly, massive expansions of gene families took place before the origins of green plants, land plants and vascular plants. Whole genome duplications (WGDs) that have occurred at least 244 times throughout the evolution of plants and ferns increase ploidy of genomes and largely impact gene family size variation within different lineages. Apart from autopolyploidy, which results from intraspecies WGD events, there are also allopolyploid species, which originate from interspecies hybrids and render gene evolution tracking challenging (Leebens-Mack et al., 2019).

### 4.1 How many *ABCE* genes do plants have and need?

As was previously mentioned, in most animal and in yeast species the *ABCE* gene family is represented by a single gene that encodes the vital ABCE1 protein. In plant kingdom, *ABCE* gene family size across different lineages is more variable. Based on the data from the public databases and our analysis we were able to reconfirm the same number of *ABCE* genes for a selection of plant species. For example, there is a single gene in *C. hirsuta* (Kougioumoutzi et al., 2013), in *Chlamydomonas reinhardtii* (Li et al., 2022), in *Citrus sinensis* and in *Theobroma cacao* (Navarro-Quiles et al., 2022). Similarly to previous studies, we reverified two genes in *Z. mays* (Pang et al., 2013), in *Solanum lycopersicum* (Ofori et al., 2018), in *Oryza sativa*, and in *Populus trichocarpa* (Navarro-Quiles et al., 2022). The same was true for five *ABCE* genes from *Brassica rapa* (Navarro-Quiles et al., 2022). Intriguingly, Zhang and others found three *ABCE* genes in *Hordeum vulgare*, whereas our study identified only two fully intact ABCE sequences with all canonical subunits (604 and 611 amino acids long) (Zhang et al., 2020). Moreover, for *C. rubella* we identified four *ABCE* genes as opposed to two sequences analyzed earlier (Navarro-Quiles et al., 2022). These discrepancies might be due to the strict filtering of non-functional protein sequences performed in our study. In addition, fast-developing sequencing technologies and implementation of novel annotation tools contribute to higher accuracy of the newer genome versions. Taken together, plant species present in our dataset possess one to eight *ABCE* genes encoding complete proteins, suggesting that plant *ABCE* genes do not comprise a single-copy gene family, but rather should be classified as a low-copy gene family.

Most plant species have experienced at least two ancient WGD events, followed by additional rounds of genome doubling in many lineages (Qiao et al., 2022). Thus, we were tempted to speculate that WGD could impact *ABCE* family size variation. However, in this study we did not notice significant correlation between the number of ancestral WGD events obtained from the literature and *ABCE* gene copy number in a species. This might be due to the rapid genome downsizing following WGD event that is leading to chromosomal rearrangements and extensive loss of duplicated copies (Wang et al., 2021). In addition, the WGD-derived duplicates are often subject to relaxed selection, meaning rapid mutation resulting in defunctionalisation (Qiao et al., 2019). For example, *Glycine max* and *A. chinensis* retained a single functional *ABCE* gene after five documented WGD events. Interestingly, the overexpression of ABCE1 in yeast causes growth inhibition (Dong et al., 2004), meaning that the amount of *ABCE* present—and therefore probably also the number of hypothetical redundant genes—is critical for the well-functioning of translation, a crucial process.

However, when higher expression of a particular gene is beneficial, its duplicate might be retained in the genome. This could be the case for the two ABCE paralogues in *Z. mays* that are located close to each other in our phylogenetic analysis (Figure 4) and share the same expression pattern profiles (Pang et al., 2013). Alternatively, as a result of faster evolution, gene duplicates may obtain novel functions or specialized expression patterns (Prince and Pickett, 2002). In Arabidopsis, AtABCE1 and AtABCE2 exhibit partial functional redundancy. In contrast to AtABCE2, which is ubiquitously expressed, AtABCE1 is mostly present in generative organs and at relatively low levels (Klepikova et al., 2016; Navarro-Quiles et al., 2022; Yu et al., 2023). This could mean an ongoing process of pseudogenization or subfunctionalization, where the paralogues acquire specific roles. There is growing evidence regarding ribosomal heterogeneity and the existence of specialized cell-type-specific ribosomes (Xue and Barna, 2012; Barna et al., 2022), suggesting that AtABCE1 is involved in the regulation of translation in generative tissues. Paralogous *ABCE* genes in plants may serve to provide specificity in fine-tuning translation and controlling cellular translatome (Gerst, 2018).

We also noticed slight positive effect of ploidy level on *ABCE* gene copy number in a species. In the future, the determinants of *ABCE* copy number can be further elucidated by including more species from diverse lineages and using statistical modelling that takes phylogenetic structuring of the data also into account. These models could also potentially incorporate other information from the species that was not used for the present study, such as whether the species is annual or perennial, their preferred mode of reproduction, or what kind of environments do they grow in.

### 4.2 In plants *ABCE* genes are prone to duplicate

In this study we analyzed 152 *ABCE* sequences from 76 plant species. This included the most well studied plant *ABCE* gene—AtABCE2, which is thought to preserve the ancestral functions of *ABCE* proteins (Navarro-Quiles et al., 2022). Phylogenetic trees of full-length *ABCE* sequences confirmed previously reported clustering into ABCE2 and ABCE1 groups for *Brassicaceae* (Navarro-Quiles et al., 2022) but also demonstrated that the multiple copies observed in several other plant lineages originate from separate duplication events. The results suggest that *Brassicaceae* and *Poaceae* families have undergone independent lineage-specific splits of the ancestral *ABCE* gene. *Pooideae*, the largest *Poaceae* subfamily that includes barley and wheat, appears to have had further duplication events and its members have additional *ABCE* genes. In addition to *Brassicaceae* and *Poaceae*, many other plant taxa have also gained *ABCE* gene copies, most likely because of more recent duplications. Interestingly, it was recently shown that one of the four *ABCE* gene copies from *Prunus dulcis*, a close relative of *P. persica*, originated from tandem duplication. *ABCE* genes share strong collinearity between these species, suggesting that this duplication preceded speciation event (Zhang et al., 2024). We can therefore postulate that in contrast to species which possess a single *ABCE* gene and are sensitive to copy number changes (Dong et al., 2004), many plants have gained additional *ABCE* copies through WGD or local duplication and likely have evolved to benefit from higher numbers of this essential translational factor. Gene copies may arise from different events including WGD, tandem- and transposon-related duplications, but the precise source of *ABCE* subfamily expansion in plants remains to be investigated.

### 4.3 Natural variation of *A. thaliana* ABCEs

Usually, essential genes are subject to strong evolutionary pressure and thus, non-synonymous SNPs in conserved regions of gene sequences are rare (Castle, 2011; Pang et al., 2016). In *AtABCE1* we found three SNPs that could potentially impact the protein’s function (Table1; Figure 5A). SNPs causing the substitutions Arg323Cys (in IP-Moz-0) and Arg572Leu (in Toufl-1) located at the Hinge domain I and II, respectively, could be of importance, since these domains are essential for NBD-twin cassette assembly in the case of ABCE1 in other organisms (Karcher et al., 2005). In addition, AtABCE1 of IP-Ezc-2 ecotype contains an amino acid substitution at position Arg86Gln, which is an exceptionally conserved site across archaea as well as eukaryotes and locates to Y-loop I. In the context of Y-loop with consensus sequence H **R** YGVNAF, the arginine residue has been shown to mediate interaction between FeS cluster domain and NBD1 in the sole *ABCE1* gene of *Pyrococcus abyssi* (Karcher et al., 2008).

The His561Leu amino acid change was reported to be present in 997 out of 1135 *A. thaliana* ecotypes (Supplementary Table S5), which suggest that histidine at this position of *AtABCE1* might be characteristic only to a small subset of ecotypes including Col-0. Thus, it seems that leucine is the most conserved residue at position 561 in *AtABCE1* among Arabidopsis ecotypes.

As expected, in *AtABCE2* gene, known to be essential for the viability of an organism (Navarro-Quiles et al., 2022; Yu et al., 2023) only four non-synonymous SNPs residing in non-conserved regions were reported among 1135 *A. thaliana* ecotypes (Supplementary Table S5). Importantly, the *AtABCE2* gene seems to be hard to mutate, since up to now there is no T-DNA homozygous line available and only one viable, hypomorphic allele has been recently isolated after ethyl methanesulfonate mutagenesis (Navarro-Quiles et al., 2022). Interestingly, the only non-synonymous SNP present in more than one ecotype in the case of *AtABCE2* is leading to Gly44Ser substitution in the FeS domain. According to the 1,001 Genomes Project database this mutation is present in 54 ecotypes, three of them were confirmed in the current study (Table 1; Figure 5; Supplementary Table S5). The same position is able to incorporate 12 different amino acid residues among other plant ABCEs studied herein, alanine, glycine and serine being the most common ones. Moreover, the SNP M379T verified only in IP-Car-1 poses a promising material for further mutational analysis as this site is highly conserved among plant ABCEs with only leucine as a rare alternative (Figure 5D; 152_pepseq_MUSCLE.fa from the associated GitHub repository).

Taken together, this study has shown the surprisingly high number of *ABCE* genes among the plant kingdom. We hypothesize that plants have developed a number of specialized ABCEs with more specific functions compared to species carrying a single copy of *ABCE* gene such as humans, fruit fly or yeast.

## Data Availability

Data relevant to this study has been made publicly available in Supplementary Materials and Github 496 repository https://github.com/jsuurvali/abce152.

## References

[B1] Andersen D. S. Leevers S. J. (2007). The essential Drosophila ATP-binding cassette domain protein, pixie, binds the 40 S ribosome in an ATP-dependent manner and is required for translation initiation. J. Biol. Chem. 282, 14752–14760. 10.1074/jbc.M701361200 17392269

[B2] Andolfo G. Ruocco M. Donato A. D. Frusciante L. Lorito M. Scala F. (2015). Genetic variability and evolutionary diversification of membrane ABC transporters in plants. BMC Plant Biol. 15, 51. 10.1186/s12870-014-0323-2 25850033 PMC4358917

[B3] Barna M. Karbstein K. Tollervey D. Ruggero D. Brar G. Greer E. L. (2022). The promises and pitfalls of specialized ribosomes. Mol. Cell. 82, 2179–2184. 10.1016/j.molcel.2022.05.035 35714581 PMC10228420

[B4] Barthelme D. Dinkelaker S. Albers S.-V. Londei P. Ermler U. Tampé R. (2011). Ribosome recycling depends on a mechanistic link between the FeS cluster domain and a conformational switch of the twin-ATPase ABCE1. Proc. Natl. Acad. Sci. U. S. A. 108, 3228–3233. 10.1073/pnas.1015953108 21292982 PMC3044390

[B5] Barthelme D. Scheele U. Dinkelaker S. Janoschka A. MacMillan F. Albers S.-V. (2007). Structural organization of essential iron-sulfur clusters in the evolutionarily highly conserved ATP-binding cassette protein ABCE1. J. Biol. Chem. 282, 14598–14607. 10.1074/jbc.M700825200 17355973

[B6] Braz A. S. K. Finnegan J. Waterhouse P. Margis R. (2004). A plant orthologue of Rnase L inhibitor (RLI) is induced in plants showing RNA interference. J. Mol. Evol. 59, 20–30. 10.1007/s00239-004-2600-4 15383904

[bib78] Cao J. Schneeberger K. Ossowski S. Günther T. Bender S. Fitz J. (2011). Whole-genome sequencing of multiple Arabidopsis thaliana populations. Nat. Genet. 43, 956–963. 10.1038/ng.911 21874002

[B7] Capella-Gutiérrez S. Silla-Martínez J. M. Gabaldón T. (2009). trimAl: a tool for automated alignment trimming in large-scale phylogenetic analyses. Bioinformatics 25, 1972–1973. 10.1093/bioinformatics/btp348 19505945 PMC2712344

[B8] Castle J. C. (2011). SNPs occur in regions with less genomic sequence conservation. PloS One 6, e20660. 10.1371/journal.pone.0020660 21674007 PMC3108954

[B9] Charif D. Lobry J. R. (2007). “SeqinR 1.0-2: a contributed package to the R project for statistical computing devoted to biological sequences retrieval and analysis,” in Structural approaches to sequence evolution: molecules, networks, populations. Editors Bastolla U. Porto M. Roman H. E. Vendruscolo M. (Berlin, Heidelberg: Biological and Medical Physics, Biomedical Engineering Springer), 207–232. 10.1007/978-3-540-35306-5_10

[B10] Dermauw W. Van Leeuwen T. (2014). The ABC gene family in arthropods: comparative genomics and role in insecticide transport and resistance. Insect biochem. Mol. Biol. 45, 89–110. 10.1016/j.ibmb.2013.11.001 24291285

[B11] Dong J. Lai R. Nielsen K. Fekete C. A. Qiu H. Hinnebusch A. G. (2004). The essential ATP-binding cassette protein RLI1 functions in translation by promoting preinitiation complex assembly. J. Biol. Chem. 279, 42157–42168. 10.1074/jbc.M404502200 15277527

[B12] Du X.-L. Wang D. Qian X.-Y. Jiang L.-Z. Chun W. Li K.-G. (2003). cDNA cloning and expression analysis of the rice (Oryza sativa L.) rnase L inhibitor. DNA Seq. 14, 295–301. 10.1080/1085566031000141162 14631652

[B13] Edgar R. C. (2004). MUSCLE: multiple sequence alignment with high accuracy and high throughput. Nucleic Acids Res. 32, 1792–1797. 10.1093/nar/gkh340 15034147 PMC390337

[B14] Edwards K. D. Fernandez-Pozo N. Drake-Stowe K. Humphry M. Evans A. D. Bombarely A. (2017). A reference genome for Nicotiana tabacum enables map-based cloning of homeologous loci implicated in nitrogen utilization efficiency. BMC Genomics 18, 448. 10.1186/s12864-017-3791-6 28625162 PMC5474855

[B15] Gan X. Hay A. Kwantes M. Haberer G. Hallab A. Ioio R. D. (2016). The *Cardamine hirsuta* genome offers insight into the evolution of morphological diversity. Nat. Plants 2, 16167–7. 10.1038/nplants.2016.167 27797353 PMC8826541

[B16] Garcia O. Bouige P. Forestier C. Dassa E. (2004). Inventory and comparative analysis of rice and Arabidopsis ATP-binding cassette (ABC) systems. J. Mol. Biol. 343, 249–265. 10.1016/j.jmb.2004.07.093 15381434

[B17] Gerst J. E. (2018). Pimp my ribosome: ribosomal protein paralogs specify translational control. Trends Genet. 34, 832–845. 10.1016/j.tig.2018.08.004 30195580

[B18] Goodstein D. M. Shu S. Howson R. Neupane R. Hayes R. D. Fazo J. (2012). Phytozome: a comparative platform for green plant genomics. Nucleic Acids Res. 40, D1178–D1186. 10.1093/nar/gkr944 22110026 PMC3245001

[B19] Graille M. Séraphin B. (2012). Surveillance pathways rescuing eukaryotic ribosomes lost in translation. Nat. Rev. Mol. Cell. Biol. 13, 727–735. 10.1038/nrm3457 23072885

[B20] Grant B. J. Skjaerven L. Yao X.-Q. (2021). The Bio3D packages for structural bioinformatics. Protein Sci. Publ. Protein Soc. 30, 20–30. 10.1002/pro.3923 PMC7737766 32734663

[B21] Howe K. L. Contreras-Moreira B. De Silva N. Maslen G. Akanni W. Allen J. (2020). Ensembl Genomes 2020—enabling non-vertebrate genomic research. Nucleic Acids Res. 48, D689-D695–D695. 10.1093/nar/gkz890 31598706 PMC6943047

[B22] Jones D. T. Taylor W. R. Thornton J. M. (1992). The rapid generation of mutation data matrices from protein sequences. Bioinformatics 8, 275–282. 10.1093/bioinformatics/8.3.275 1633570

[B23] Kärblane K. Gerassimenko J. Nigul L. Piirsoo A. Smialowska A. Vinkel K. (2015). ABCE1 is a highly conserved RNA silencing suppressor. PloS One 10, e0116702. 10.1371/journal.pone.0116702 25659154 PMC4319951

[B24] Karcher A. Büttner K. Märtens B. Jansen R.-P. Hopfner K.-P. (2005). X-ray structure of RLI, an essential twin cassette ABC ATPase involved in ribosome biogenesis and HIV capsid assembly. Struct. Lond. Engl. 13, 649–659. 10.1016/j.str.2005.02.008 15837203

[B25] Karcher A. Schele A. Hopfner K.-P. (2008). X-ray structure of the complete ABC enzyme ABCE1 from *Pyrococcus abyssi* . J. Biol. Chem. 283, 7962–7971. 10.1074/jbc.M707347200 18160405

[B26] Katoh K. Standley D. M. (2013). MAFFT multiple sequence alignment software version 7: improvements in performance and usability. Mol. Biol. Evol. 30, 772–780. 10.1093/molbev/mst010 23329690 PMC3603318

[B27] Kispal G. Sipos K. Lange H. Fekete S. Bedekovics T. Janáky T. (2005). Biogenesis of cytosolic ribosomes requires the essential iron–sulphur protein Rli1p and mitochondria. EMBO J. 24, 589–598. 10.1038/sj.emboj.7600541 15660134 PMC548650

[B28] Klepikova A. V. Kasianov A. S. Gerasimov E. S. Logacheva M. D. Penin A. A. (2016). A high resolution map of the *Arabidopsis thaliana* developmental transcriptome based on RNA-seq profiling. Plant J. 88, 1058–1070. 10.1111/tpj.13312 27549386

[B29] Kougioumoutzi E. Cartolano M. Canales C. Dupré M. Bramsiepe J. Vlad D. (2013). SIMPLE LEAF3 encodes a ribosome-associated protein required for leaflet development in *Cardamine hirsuta* . Plant J. 73, 533–545. 10.1111/tpj.12072 23145478

[B30] Kourelis J. Kaschani F. Grosse-Holz F. M. Homma F. Kaiser M. van der Hoorn R. A. L. (2019). A homology-guided, genome-based proteome for improved proteomics in the alloploid *Nicotiana benthamiana* . BMC Genomics 20, 722. 10.1186/s12864-019-6058-6 31585525 PMC6778390

[B32] Leebens-Mack J. H. Barker M. S. Carpenter E. J. Deyholos M. K. Gitzendanner M. A. Graham S. W. (2019). One thousand plant transcriptomes and the phylogenomics of green plants. Nature 574, 679–685. 10.1038/s41586-019-1693-2 31645766 PMC6872490

[B33] Leigh J. W. Bryant D. (2015). Popart: full-feature software for haplotype network construction. Methods Ecol. Evol. 6, 1110–1116. 10.1111/2041-210X.12410

[B34] Letunic I. Bork P. (2007). Interactive Tree of Life (iTOL): an online tool for phylogenetic tree display and annotation. Bioinforma. Oxf. Engl. 23, 127–128. 10.1093/bioinformatics/btl529 17050570

[B35] Letunic I. Bork P. (2019). Interactive Tree of Life (iTOL) v4: recent updates and new developments. Nucleic Acids Res. 47, W256-W259–W259. 10.1093/nar/gkz239 30931475 PMC6602468

[B36] Li X. Li X. Yang X. Lan C. Huang Y. Jia B. (2022). Identification and characterization of ATP-binding cassette transporters in *Chlamydomonas reinhardtii* . Mar. Drugs. 20, 603. 10.3390/md20100603 36286426 PMC9605142

[B37] Liu S. Li Q. Liu Z. (2013). Genome-wide identification, characterization and phylogenetic analysis of 50 catfish ATP-binding cassette (ABC) transporter genes. PloS One 8, e63895. 10.1371/journal.pone.0063895 23696857 PMC3655950

[B38] Lu H. Xu Y. Cui F. (2016). Phylogenetic analysis of the ATP-binding cassette transporter family in three mosquito species. Pestic. Biochem. Physiol. 132, 118–124. 10.1016/j.pestbp.2015.11.006 27521922

[B39] Lüdecke D. (2018). Ggeffects: tidy data frames of marginal effects from regression models. J. Open Source Softw. 3 (26), 772. 10.21105/joss.00772

[B40] Madeira F. Park Y. M. Lee J. Buso N. Gur T. Madhusoodanan N. (2019). The EMBL-EBI search and sequence analysis tools APIs in 2019. Nucleic Acids Res. 47, W636-W641–W641. 10.1093/nar/gkz268 30976793 PMC6602479

[B41] Maeda T. Lee J. M. Miyagawa Y. Koga K. Kawaguchi Y. Kusakabe T. (2005). Cloning and characterization of a ribonuclease L inhibitor from the silkworm, *Bombyx mori* . Bombyx Mori. DNA Seq. 16, 21–27. 10.1080/10425170400028871 16040343

[B42] Mancera-Martínez E. Querido J. B. Valasek L. S. Simonetti A. Hashem Y. (2017). ABCE1: a special factor that orchestrates translation at the crossroad between recycling and initiation. RNA Biol. 14, 1279–1285. 10.1080/15476286.2016.1269993 28498001 PMC5711452

[B43] Minh B. Q. Schmidt H. A. Chernomor O. Schrempf D. Woodhams M. D. von Haeseler A. (2020). IQ-TREE 2: new models and efficient methods for phylogenetic inference in the genomic era. Mol. Biol. Evol. 37 (5), 1530–1534. 10.1093/molbev/msaa015 32011700 PMC7182206

[B44] Mõttus J. Maiste S. Eek P. Truve E. Sarmiento C. (2021). Mutational analysis of *Arabidopsis thaliana* ABCE2 identifies important motifs for its RNA silencing suppressor function. Plant Biol. 23, 21–31. 10.1111/plb.13193 33040451 PMC7839781

[B45] Navarro-Quiles C. Mateo-Bonmatí E. Candela H. Robles P. Martínez-Laborda A. Fernández Y. (2022). The Arabidopsis ATP-Binding Cassette E protein ABCE2 is a conserved component of the translation machinery. Front. Plant Sci. 13, 1009895. 10.3389/fpls.2022.1009895 36325553 PMC9618717

[B46] Navarro-Quiles C. Mateo-Bonmatí E. Micol J. L. (2018). ABCE proteins: from molecules to development. Front. Plant Sci. 9, 1125. 10.3389/fpls.2018.01125 30127795 PMC6088178

[B47] Nürenberg E. Tampé R. (2013). Tying up loose ends: ribosome recycling in eukaryotes and archaea. Trends biochem. Sci. 38, 64–74. 10.1016/j.tibs.2012.11.003 23266104

[B48] Nürenberg‐Goloub E. Kratzat H. Heinemann H. Heuer A. Kötter P. Berninghausen O. (2020). Molecular analysis of the ribosome recycling factor ABCE1 bound to the 30S post-splitting complex. EMBO J. n/a 39, e103788. 10.15252/embj.2019103788 PMC7196836 32064661

[B49] Ofori P. A. Geisler M. Di Donato M. Pengchao H. Otagaki S. Matsumoto S. (2018). Tomato ATP-binding cassette transporter SlABCB4 is involved in auxin transport in the developing fruit. Plants Basel Switz. 7, 65. 10.3390/plants7030065 PMC6161087 30104476

[B50] Pang E. Wu X. Lin K. (2016). Different evolutionary patterns of SNPs between domains and unassigned regions in human protein-coding sequences. Mol. Genet. Genomics. 291, 1127–1136. 10.1007/s00438-016-1170-7 26833483 PMC4875946

[B51] Pang K. Li Y. Liu M. Meng Z. Yu Y. (2013). Inventory and general analysis of the ATP-binding cassette (ABC) gene superfamily in maize (*Zea mays* L.). Gene 526, 411–428. 10.1016/j.gene.2013.05.051 23747399

[B52] Pedersen T. (2024). Patchwork: the composer of plots. R. package version 1.2.0. Available at: https://github.com/thomasp85/patchwork.

[B53] Petersen B. O. Jørgensen B. Albrechtsen M. (2004). Isolation and RNA silencing of homologues of the Rnase L inhibitor in Nicotiana species. Plant Sci. 167, 1283–1289. 10.1016/j.plantsci.2004.06.030

[B54] Pinheiro J. Bates D. R Core Team (2023). Nlme: linear and nonlinear mixed effects models. R. package version 3, 1–164. Available at: https://CRAN.R-project.org/package=nlme.

[B55] Pinheiro J. C. Bates D. M. (2000) Mixed-effects models in S and S-plus. New York: Springer. 10.1007/b98882

[B56] Prince V. E. Pickett F. B. (2002). Splitting pairs: the diverging fates of duplicated genes. Nat. Rev. Genet. 3, 827–837. 10.1038/nrg928 12415313

[B57] Qiao X. Li Q. Yin H. Qi K. Li L. Wang R. (2019). Gene duplication and evolution in recurring polyploidization–diploidization cycles in plants. Genome Biol. 20, 38. 10.1186/s13059-019-1650-2 30791939 PMC6383267

[B58] Qiao X. Zhang S. Paterson A. H. (2022). Pervasive genome duplications across the plant tree of life and their links to major evolutionary innovations and transitions. Comput. Struct. Biotechnol. J. 20, 3248–3256. 10.1016/j.csbj.2022.06.026 35782740 PMC9237934

[B59] R Core Team (2023) R: a language and environment for statistical computing. Vienna, Austria: R Foundation for Statistical Computing. Available at: https://www.r-project.org/.

[B60] Sarmiento C. Nigul L. Kazantseva J. Buschmann M. Truve E. (2006). AtRLI2 is an endogenous suppressor of RNA silencing. Plant Mol. Biol. 61, 153–163. 10.1007/s11103-005-0001-8 16786298

[B61] Soubrier J. Steel M. Lee M. S. Y. Der Sarkissian C. Guindon S. Ho S. Y. W. (2012). The influence of rate heterogeneity among sites on the time dependence of molecular rates. Mol. Biol. Evol. 29 (11), 3345–3358. 10.1093/molbev/mss140 22617951

[B63] Stöver B. C. Müller K. F. (2010). TreeGraph 2: combining and visualizing evidence from different phylogenetic analyses. BMC Bioinforma. 11 (7), 7. 10.1186/1471-2105-11-7 PMC2806359 20051126

[B64] Strunk B. S. Novak M. N. Young C. L. Karbstein K. (2012). A translation-like cycle is a quality control checkpoint for maturing 40S ribosome subunits. Cell. 150, 111–121. 10.1016/j.cell.2012.04.044 22770215 PMC3615461

[B65] Tamura K. Stecher G. Kumar S. (2021). MEGA11: molecular evolutionary Genetics analysis version 11. Mol. Biol. Evol. 38 (7), 3022–3027. 10.1093/molbev/msab120 33892491 PMC8233496

[B66] Toompuu M. Kärblane K. Pata P. Truve E. Sarmiento C. (2016). ABCE1 is essential for S phase progression in human cells. Tex 15, 1234–1247. 10.1080/15384101.2016.1160972 PMC4889273 26985706

[B67] Verrier P. J. Bird D. Burla B. Dassa E. Forestier C. Geisler M. (2008). Plant ABC proteins—a unified nomenclature and updated inventory. Trends Plant Sci. 13, 151–159. 10.1016/j.tplants.2008.02.001 18299247

[B68] Wang X. Morton J. A. Pellicer J. Leitch I. J. Leitch A. R. (2021). Genome downsizing after polyploidy: mechanisms, rates and selection pressures. Plant J. 107, 1003–1015. 10.1111/tpj.15363 34077584

[B69] Wang X. Wang H. Wang J. Sun R. Wu J. Liu S. (2011). The genome of the mesopolyploid crop species *Brassica rapa* . Nat. Genet. 43, 1035–1039. 10.1038/ng.919 21873998

[B70] Weigel D. Mott R. (2009). The 1001 genomes project for *Arabidopsis thaliana* . Genome Biol. 10, 107. 10.1186/gb-2009-10-5-107 19519932 PMC2718507

[B71] Wickham H. (2016) ggplot2: elegant graphics for data analysis. New York: Springer-Verlag. Available at: https://ggplot2.tidyverse.org.

[B72] Xue S. Barna M. (2012). Specialized ribosomes: a new frontier in gene regulation and organismal biology. Nat. Rev. Mol. Cell. Biol. 13, 355–369. 10.1038/nrm3359 22617470 PMC4039366

[B73] Yarunin A. Panse V. G. Petfalski E. Dez C. Tollervey D. Hurt E. C. (2005). Functional link between ribosome formation and biogenesis of iron–sulfur proteins. EMBO J. 24, 580–588. 10.1038/sj.emboj.7600540 15660135 PMC548649

[B74] Yu S.-X. Hu L.-Q. Yang L.-H. Zhang T. Dai R.-B. Zhang Y.-J. (2023). RLI2 regulates Arabidopsis female gametophyte and embryo development by facilitating the assembly of the translational machinery. Cell. Rep. 42, 112741. 10.1016/j.celrep.2023.112741 37421624

[B75] Zhang D. Yu Z. Zeng B. Liu X. (2024). Genome-wide analysis of the ABC gene family in almond and functional predictions during flower development, freezing stress, and salt stress. BMC Plant Biol. 24 (1), 12. 10.1186/s12870-023-04698-7 38163883 PMC10759767

[B76] Zhang Z. Tong T. Fang Y. Zheng J. Zhang X. Niu C. (2020). Genome-wide identification of barley ABC genes and their expression in response to abiotic stress treatment. Plants Basel Switz. 9, 1281. 10.3390/plants9101281 PMC7599588 32998428

[B77] Zhao Z. Fang L. L. Johnsen R. Baillie D. L. (2004). ATP-binding cassette protein E is involved in gene transcription and translation in *Caenorhabditis elegans* . Biochem. Biophys. Res. Commun. 323, 104–111. 10.1016/j.bbrc.2004.08.068 15351708

