# Supplementary material for: Phylogenetic insight into ABCE gene subfamily in plants
Source: Front Genet. 2024 Jun 7;15:1408665. doi: 10.3389/fgene.2024.1408665 (PMC11190730; doi:10.3389/fgene.2024.1408665)
Supplement: Supplementary file 1 [file DataSheet2.PDF]

Supplementary Table S2. List of the 76 plant species used within the *ABCE* phylogenetic analysis. Number of plant *ABCE* genes identified per species, database used, genome assembly version and genome ploidy level are depicted.

No	Latin name	English name	Abbreviated name	No of genes	Ploidy	Dataset	Assembly version
1	*Actinidia chinensis Red5*	golden kiwifruit	Ach	1	diploid	Ensembl	Red5_PS1_1.69.0
2	*Aegilops tauschii*	Tausch's goatgrass, rough-spike hard grass	Ata	2	diploid	Ensembl	Aet_v4.0
3	*Amaranthus hypochondriacus*	Prince's-feather	Ahy	2	diploid	Phytozome	v1.0
4	*Amborella trichopoda*	amborella	Atr	2	diploid endosperm??	Phytozome	v1.0
5	*Ananas comosus*	pineapple	Acom	1	diploid	Phytozome	v3
6	*Aquilegia coerulea*	colorado blue columbine	Acoe	2	diploid	Phytozome	v3.1
7	*Arabidopsis halleri*	-	Aha	2	diploid	Phytozome	v2.1.0
8	*Arabidopsis lyrata*	-	Aly	2	diploid	Phytozome	v2.1
9	*Arabidopsis thaliana*	thale cress	Ath	2	diploid	Phytozome	TAIR10
10	*Beta vulgaris*	beet	Bvu	2	diploid	Ensembl	RefBeet-1.2.2
11	*Boechera stricta*	drummond's rockcress	Bstricta	1	diploid	Phytozome	v1.2
12	*Brachypodium distachyon*	purple false brome, stiff brome	Bdi	5	diploid	Phytozome	V3.1
13	*Brachypodium stacei*	-	Bstacei	4	diploid	Phytozome	v1.1
14	*Brassica napus*	rapeseed, oilseed rape	Bna	8	allotetraploid	Ensembl	AST_PRJEB5043_v1
15	*Brassica oleracea*	wild cabbage	Bol	3	diploid	Phytozome	v1.0
16	*Brassica rapa*	field mustard	Bra	5	diploid	Phytozome	FPsc v1.3
17	*Capsella grandiflora*	grand shepherd's-purse	Cgr	2	diploid	Phytozome	v1.1
18	*Capsella rubella*	pink shepherd's-purse	Cru	4	diploid	Phytozome	v1.0
19	*Cardamine hirsuta*	hairy bittercress	Chi	1	diploid	Gan et al., 2016	MIPS_CARH_v3.8
20	*Chlamydomonas reinhardtii*	green alga	Cre	1	haploid	Phytozome	v5.5
21	*Citrus clementina*	clementine	Ccl	2	diploid	Phytozome	v1.0
22	*Citrus sinensis*	sweet orange	Csi	1	diploid	Phytozome	v1.1
23	*Corchorus capsularis*	white jute	Cca	1	diploid	Ensembl	CCACVL1_1.0
24	*Cucumis sativus*	cucumber	Csa	1	diploid	Phytozome	v1.0
25	*Daucus carota*	wild carrot, bird's nest, bishop's lace	Dca	1	diploid	Phytozome	v2.0
26	*Dioscorea rotundata*	white yam, Guinea yam	Dro	1	diploid	Ensembl	TDr96_F1_Pseudo_Chromosome_v1.0
27	*Erythranthe guttata*	seep monkeyflower	Egu	2	diploid	Phytozome	v2.0
28	*Eucalyptus grandis*	flooded gum, rose gum	Egr	1	diploid	Phytozome	v2.0
29	*Eutrema salsugineum*	saltwater cress	Esa	2	diploid	Phytozome	v1.0
30	*Glycine max*	soybean	Gma	1	functionally diploid	Phytozome	v2.0
31	*Gossypium raimondii*	cotton plant endemic to Peru	Gra	1	diploid	Phytozome	v2.1
32	*Helianthus annuus*	common sunflower	Han	2	diploid	Ensembl	HanXRQr1.0
33	*Hordeum vulgare*	barley	Hvu	2	diploid	Ensembl	IBSC_v2
34	*Kalanchoe fedtschenkoi*	kalanchoë	Kfe	1	diploid	Phytozome	v1.1
35	*Kalanchoe laxiflora*	Milky Widow's Thrill	Kla	2	diploid	Phytozome	v1.1
36	*Linum usitatissimum*	common flax, linseed	Lus	2	diploid	Phytozome	v1.0
37	*Lupinus angustifolius*	narrow-leaved blue lupine	Lan	2	diploid	Ensembl	LupAngTanjil_v1.0
38	*Manihot esculenta*	cassava, manioc, yuca	Mes	2	diploid	Phytozome	v6.1
39	*Marchantia polymorpha*	common liverwort, umbrella liverwort	Mpo	1	haploid/diploid	Phytozome	v3.1
40	*Medicago truncatula*	barrelclover, strong-spined medick, barrel medic	Mtr	3	diploid	Phytozome	Mt4.0v1
41	*Micromonas sp. RCC299*	green alga	Msp	1	haploid	Phytozome	v3.0
42	*Musa acuminata*	banana native to Southeast Asia	Mac	3	double haploid	Phytozome	v1
43	*Nicotiana attenuata*	coyote tobacco	Nat	1	diploid	Ensembl	NIATTr2
44	*Nicotiana benthamiana*	tobacco	Nbe	2	allotetraploid	Kourelis et al. 2019	v1.0.1
45	*Nicotiana tabacum*	cultivated tobacco	Nta	1	allotetraploid	solgenomics.net	TN90 protein sequences
46	*Oropetium thomaeum*	-	Oth	1	diploid	Phytozome	v1.0
47	*Oryza brachyantha*	rice grass native to tropical Africa	Obr	2	diploid	Ensembl	v1.4b
48	*Oryza glaberrima*	african rice	Ogl	2	diploid	Ensembl	V1
49	*Oryza longistaminata*	african wild rice	Olo	2	diploid	Ensembl	v1.0
50	*Oryza sativa Japonica Group*	sinica rice	OsaJ	2	diploid	Phytozome	v7_JGI

No	Latin name	English name	Abbreviated name	No of genes	Ploidy	Dataset	Assembly version
51	*Ostreococcus lucimarinus*	green alga	Olu	2	haploid	Phytozome	v2.0
52	*Panicum hallii ecotype FIL2*	hall's panicgrass	Pha	2	diploid	Ensembl	PHallii_v3.1
53	*Panicum virgatum*	switchgrass	Pvi	4	allotetraploid	Phytozome	v1.1
54	*Phaseolus vulgaris*	common bean, French bean	Pvu	1	diploid	Phytozome	v2.1
55	*Physcomitrella patens*	spreading earthmoss	Ppa	2	haploid/diploid	Phytozome	v3.3
56	*Populus trichocarpa*	black cottonwood, California poplar	Ptr	2	diploid	Phytozome	v3.0
57	*Prunus persica*	peach	Ppe	3	diploid	Phytozome	v2.1
58	*Ricinus communis*	castor bean	Rco	1	diploid	Phytozome	v0.1
59	*Salix purpurea*	purple willow	Spu	2	diploid	Phytozome	v1.0
60	*Setaria italica*	foxtail millet	Sit	2	diploid	Phytozome	v2.2
61	*Setaria viridis*	green foxtail, wild foxtail millet	Svi	2	diploid	Phytozome	v1.1
62	*Solanum lycopersicum*	tomato	Sly	1	diploid	Phytozome	iTAG2.4
63	*Solanum tuberosum*	potato	Stu	1	diploid/ tetraploid	Phytozome	v4.03
64	*Sorghum bicolor*	sorghum, great millet	Sbi	2	diploid	Phytozome	v3.1.1
65	*Sphagnum fallax*	flat-topped bogmoss	Sfa	2	haploid	Phytozome	v0.5
66	*Spirodela polyrhiza*	common duckmeat	Spo	1	diploid	Phytozome	v2
67	*Zea mays Ensembl-18*	maize, corn	Zmays	2	diploid	Phytozome	AGPv3
68	*Zostera marina*	common eelgrass, seawrack	Zmarina	1	diploid	Phytozome	v2.2
69	*Theobroma cacao*	cacao tree	Tca	1	diploid	Phytozome	v1.1
70	*Trifolium pratense*	red clover	Tpr	2	diploid	Phytozome	v2
71	*Triticum aestivum*	common wheat, bread wheat	Tae	8	allohexaploid	Ensembl	IWGSC
72	*Triticum dicoccoides*	emmer wheat, hulled wheat	Tdi	4	allotetraploid	Ensembl	WEWSeq_v.1.0
73	*Vigna angularis*	adzuki bean, English red mung bean	Van	1	diploid	Ensembl	Vigan1.1
74	*Vigna radiata*	mung bean	Vra	1	diploid	Ensembl	Vradiata_ver6
75	*Vitis vinifera*	common grape vine	Vvi	1	diploid	Phytozome	Genoscope.12X
76	*Volvox carteri*	colonial green alga	Vca	1	haploid/diploid	Phytozome	v2.1
