# Supplementary material for: Phylogenetic insight into ABCE gene subfamily in plants
Source: Front Genet. 2024 Jun 7;15:1408665. doi: 10.3389/fgene.2024.1408665 (PMC11190730; doi:10.3389/fgene.2024.1408665)
Supplement: Supplementary file 2 [file DataSheet4.PDF]

Supplementary Table S4. List of the 152 *ABCE* genes identified from 76 plant species together with protein length and amino acid sequence similarity to AtABCE2.

No	Latin species name	Species acronym in this study	Locus/gene/transcript name	Name used within this study	Peptide length	Amino acid similarity with AtABCE2
1	*Actinidia chinensis Red5*	ach	*CEY00_Acc10365; PSS21323*	ach_Acc10365:PSS21323	605	95.9%
2	*Aegilops tauschii*	ata	*AET2Gv21244700.1*	ata_AET2Gv21244700.1	604	92.4%
3	*Aegilops tauschii*	ata	*AET4Gv20370200.1*	ata_AET4Gv20370200.1	604	93.2%
4	*Amaranthus hypochondriacus*	ahy	*AHYPO_014383-AR*	ahy_AHYPO_014383	605	95.0%
5	*Amaranthus hypochondriacus*	ahy	*AHYPO_015513-AR*	ahy_AHYPO_015513	605	95.4%
6	*Amborella trichopoda*	atr	*evm_27.model.AmTr_v1.0_scaffold00066.5*	atr_scaffold00066.5	606	95.5%
7	*Amborella trichopoda*	atr	*evm_27.model.AmTr_v1.0_scaffold00226.4*	atr_scaffold00226.4	605	89.4%
8	*Ananas comosus*	acom	*Aco010811.1*	acom_Aco010811.1	605	95.5%
9	*Aquilegia coerulea*	acoe	*Aqcoe4G278700.1*	acoe_Aqcoe4G278700.1	604	96.4%
10	*Aquilegia coerulea*	acoe	*Aqcoe7G097700.1*	acoe_Aqcoe7G097700.1	590	85.4%
11	*Arabidopsis halleri*	aha	*Ah3G15670.1*	aha_Ah3G15670.1	603	88.1%
12	*Arabidopsis halleri*	aha	*Ah7G23380.1*	aha_Ah7G23380.1	605	99.7%
13	*Arabidopsis lyrata*	aly	*AL3G25580.t1*	aly_AL3G25580	581	87.6%
14	*Arabidopsis lyrata*	aly	*AL486U10010.t1*	aly_AL486U10010	605	99.5%
15	*Arabidopsis thaliana*	ath	*AT3G13640.1; AtABCE1*	AtABCE1	603	87.1%
**16**	***Arabidopsis thaliana***	**ath**	***AT4G19210.1; AtABCE2***	**AtABCE2**	**605**	**100.0%**
17	*Beta vulgaris*	bvu	*BVRB_1g018680; KMT00039*	bvu_BVRB_1g018680:KMT00039	605	93.4%
18	*Beta vulgaris*	bvu	*BVRB_9g216910; KMT00386*	bvu_BVRB_9g216910:KMT00386	605	94.7%
19	*Boechera stricta*	bstricta	*Bostr.30275s0405.1*	bstricta_Bostr.30275s0405.1	605	99.0%
20	*Brachypodium distachyon*	bdi	*Bradi3g10390.1*	bdi_Bradi3g10390	603	78.6%
21	*Brachypodium distachyon*	bdi	*Bradi3g33470.1*	bdi_Bradi3g33470	603	88.3%
22	*Brachypodium distachyon*	bdi	*Bradi4g16800.1*	bdi_Bradi4g16800	604	93.9%
23	*Brachypodium distachyon*	bdi	*Bradi5g26531.1*	bdi_Bradi5g26531	604	92.7%
24	*Brachypodium distachyon*	bdi	*Bradi5g26541.1*	bdi_Bradi5g26541	604	93.4%
25	*Brachypodium stacei*	bstacei	*Brast03G199600.1*	bstacei_Brast03G199600	608	88.5%
26	*Brachypodium stacei*	bstacei	*Brast09G261100.1*	bstacei_Brast09G261100	604	93.0%
27	*Brachypodium stacei*	bstacei	*Brast09G261200.1*	bstacei_Brast09G261200	604	93.0%
28	*Brachypodium stacei*	bstacei	*Brast10G121800.1*	bstacei_Brast10G121800	604	93.9%
29	*Brassica napus*	bna	*BnaA01g37290D; CDY60809*	bna_GSBRNA2T00030095001:CDY60809	603	88.1%
30	*Brassica napus*	bna	*BnaA03g43940D; CDX98609*	bna_GSBRNA2T00106646001:CDX98609	605	99.0%
31	*Brassica napus*	bna	*BnaC01g11640D; CDX99388*	bna_GSBRNA2T00107708001:CDX99388	605	98.2%
32	*Brassica napus*	bna	*BnaC07g35780D; CDY01588*	bna_GSBRNA2T00112634001:CDY01588	605	99.0%
33	*Brassica napus*	bna	*BnaC05g46060D; CDY05197*	bna_GSBRNA2T00120003001:CDY05197	605	98.0%
34	*Brassica napus*	bna	*BnaC03g38060D; CDX75723*	bna_GSBRNA2T00123640001:CDX75723	603	85.4%
35	*Brassica napus*	bna	*BnaA08g09150D; CDX76485*	bna_GSBRNA2T00126902001:CDX76485	605	98.8%
36	*Brassica napus*	bna	*BnaA01g09970D; CDX78930*	bna_GSBRNA2T00131424001:CDX78930	605	98.0%
37	*Brassica oleracea*	bol	*Bol009339*	bol_Bol009339	605	98.2%
38	*Brassica oleracea*	bol	*Bol024386*	bol_Bol024386	605	99.0%
39	*Brassica oleracea*	bol	*Bol035541*	bol_Bol035541	603	85.4%
40	*Brassica rapa*	bra	*Brara.A01027.1*	bra_Brara.A01027	605	98.0%
41	*Brassica rapa*	bra	*Brara.A03248.1*	bra_Brara.A03248	603	88.1%
42	*Brassica rapa*	bra	*Brara.C03483.1*	bra_Brara.C03483	603	85.4%
43	*Brassica rapa*	bra	*Brara.H01017.1*	bra_Brara.H01017	605	98.8%
44	*Brassica rapa*	bra	*Brara.K01755.1*	bra_Brara.K01755	605	99.0%
45	*Capsella grandiflora*	cgr	*Cagra.11598s0003.1*	cgr_Cagra.11598s0003	605	99.2%
46	*Capsella grandiflora*	cgr	*Cagra.1189s0042.1*	cgr_Cagra.1189s0042	604	86.2%
47	*Capsella rubella*	cru	*Carubv10007560m*	cru_Carubv10007560m.g	605	99.2%
48	*Capsella rubella*	cru	*Carubv10013264m*	cru_Carubv10013264m.g	603	87.9%
49	*Capsella rubella*	cru	*Carubv10015171m*	cru_Carubv10015171m.g	604	86.4%
50	*Capsella rubella*	cru	*Carubv10015991m*	cru_Carubv10015991m.g	603	87.9%
51	*Cardamine hirsuta*	chi	*CARHR226120; ChRLI2*	chi_RLI2	605	99.3%
52	*Chlamydomonas reinhardtii*	cre	*Cre09.g409350.t1.2*	cre_Cre09.g409350	618	86.7%
53	*Citrus clementina*	ccl	*Ciclev10011325m*	ccl_Ciclev10011325m	605	95.0%
54	*Citrus clementina*	ccl	*Ciclev10011326m*	ccl_Ciclev10011326m	605	95.0%
55	*Citrus sinensis*	csi	*orange1.1g040300m*	csi_orange1.1g040300m.g	605	94.9%
56	*Corchorus capsularis*	cca	*CCACVL1_13469; OMO79727*	cca_OMO79727	605	94.2%
57	*Cucumis sativus*	csa	*Cucsa.327120.1*	csa_Cucsa.327120	605	95.9%
58	*Daucus carota*	dca	*DCAR_019007*	dca_DCAR_019007	605	94.5%
59	*Dioscorea rotundata*	dro	*Dr09618; Dr09618.1*	dro_Dr09618.1	625	93.7%
60	*Erythranthe guttata*	egu	*Migut.A00525.1*	egu_Migut.A00525	605	95.0%
61	*Erythranthe guttata*	egu	*Migut.N01668.1*	egu_Migut.N01668	604	85.4%
62	*Eucalyptus grandis*	egr	*Eucgr.D02563.1*	egr_Eucgr.D02563	605	94.9%
63	*Eutrema salsugineum*	esa	*Thhalv10002453m*	esa_Thhalv10002453m	599	86.6%
64	*Eutrema salsugineum*	esa	*Thhalv10024707m*	esa_Thhalv10024707m	605	98.5%
65	*Glycine max*	gma	*Glyma.11G090900.1*	gma_Glyma.11G090900.1.p	606	95.4%
66	*Gossypium raimondii*	gra	*Gorai.004G177600.2*	gra_orai.004G177600	605	96.0%
67	*Helianthus annuus*	han	*OTG18841*	han_OTG18841	605	94.7%
68	*Helianthus annuus*	han	*OTG19405*	han_OTG19405	605	94.5%
69	*Hordeum vulgare*	hvu	*HORVU2Hr1G123150.1*	hvu_HORVU2Hr1G123150.1	611	92.2%
70	*Hordeum vulgare*	hvu	*HORVU4Hr1G038380.2*	hvu_HORVU4Hr1G038380.2	604	93.4%

No	Latin species name	Species acronym in this study	Locus/gene/transcript name	Name used within this study	Peptide length	Amino acid similarity with AtABCE2
71	*Kalanchoe fedtschenkoi*	kfe	*Kaladp0011s0742.1*	kfe_Kaladp0011s0742	605	94.7%
72	*Kalanchoe laxiflora*	kla	*Kalax.0125s0034.1*	kla_Kalax.0125s0034	605	94.7%
73	*Kalanchoe laxiflora*	kla	*Kalax.0585s0005.1*	kla_Kalax.0585s0005	605	94.7%
74	*Linum usitatissimum*	lus	*Lus10033309*	lus_Lus10033309.g	605	94.9%
75	*Linum usitatissimum*	lus	*Lus10034771*	lus_Lus10034771.g	605	94.9%
76	*Lupinus angustifolius*	lan	*TanjilG_20581; OIV92919*	lan_TanjilG_20581:OIV92919	606	94.9%
77	*Lupinus angustifolius*	lan	*TanjilG_25459; OIV94397*	lan_TanjilG_25459:OIV94397	606	94.5%
78	*Manihot esculenta*	mes	*Manes.04G077300.1*	mes_Manes.04G077300	605	95.7%
79	*Manihot esculenta*	mes	*Manes.04G077500.1*	mes_Manes.04G077500	605	95.7%
80	*Marchantia polymorpha*	mpo	*Mapoly0081s0022.2*	mpo_Mapoly0081s0022	605	94.5%
81	*Medicago truncatula*	mtr	*Medtr1g025075.1*	mtr_Medtr1g025075	607	92.6%
82	*Medicago truncatula*	mtr	*Medtr1g114170.1*	mtr_Medtr1g114170	606	94.5%
83	*Medicago truncatula*	mtr	*Medtr4g007890.1*	mtr_Medtr4g007890	601	88.5%
84	*Micromonas sp. RCC299*	msp	*64787*	msp_64787	614	85.6%
85	*Musa acuminata*	mac	*GSMUA_Achr10T22260_001*	mac_GSMUA_Achr10G22260_001	605	95.7%
86	*Musa acuminata*	mac	*GSMUA_Achr7T14480_001*	mac_GSMUA_Achr7G14480_001	605	95.5%
87	*Musa acuminata*	mac	*GSMUA_Achr7T14490_001*	mac_GSMUA_Achr7G14490_001	605	95.5%
88	*Nicotiana attenuata*	nat	*ABCE2; OIT28361*	nat_OIT28361	606	95.7%
89	*Nicotiana benthamiana*	nbe	*Niben101Scf08193g00024.1; NbABCE1*	nbe_NbABCE1	606	95.0%
90	*Nicotiana benthamiana*	nbe	*Niben101Scf02548g06001.1; NbABCE2*	nbe_NBABCE2	606	95.4%
91	*Nicotiana tabacum*	nta	*mRNA_25775*	nta_mRNA_25775	606	95.5%
92	*Oropetium thomaeum*	oth	*Oropetium_20150105_20507A*	oth_Oropetium_20150105_20507	604	95.5%
93	*Oryza brachyantha*	obr	*OB02G20970; OB02G20970.1*	obr_OB02G20970.1	607	80.1%
94	*Oryza brachyantha*	obr	*OB11G22540; OB11G22540.1*	obr_OB11G22540.1	604	95.0%
95	*Oryza glaberrima*	ogl	*ORGLA02G0111500.1*	ogl_ORGLA02G0111500.1	607	81.3%
96	*Oryza glaberrima*	ogl	*ORGLA10G0095700.1*	ogl_ORGLA10G0095700.1	593	84.1%
97	*Oryza longistaminata*	olo	*KN538870.1_FG005*	olo_KN538870.1_FG005	626	81.2%
98	*Oryza longistaminata*	olo	*KN539855.1_FG003*	olo_KN539855.1_FG003	676	81.5%
99	*Oryza sativa Japonica Group*	osaJ	*LOC_Os02g18180.1*	osaJ_LOC_Os02g18180	608	81.3%
100	*Oryza sativa Japonica Group*	osaJ	*LOC_Os11g34350.1*	osaJ_LOC_Os11g34350	604	95.0%
101	*Ostreococcus lucimarinus*	olu	*27256*	olu_eugene.1300010278_27256	611	83.1%
102	*Ostreococcus lucimarinus*	olu	*29654*	olu_eugene.2100010028_29654	611	83.1%
103	*Panicum hallii ecotype FIL2*	pha	*PAHAL_1G128700; PAN05285*	pha_PAHAL_1G128700:PAN05285	604	79.8%
104	*Panicum hallii ecotype FIL2*	pha	*PAHAL_8G202200; PAN42922*	pha_PAHAL_8G202200:PAN42922	604	95.5%
105	*Panicum virgatum*	pvi	*Pavir.Aa03530.1*	pvi_Pavir.Aa03530	603	84.0%
106	*Panicum virgatum*	pvi	*Pavir.Ha01484.1*	pvi_Pavir.Ha01484	604	95.5%
107	*Panicum virgatum*	pvi	*Pavir.Hb00700.1*	pvi_Pavir.Hb00700	604	95.5%
108	*Panicum virgatum*	pvi	*Pavir.J25378.1*	pvi_Pavir.J25378	603	84.4%
109	*Phaseolus vulgaris*	pvu	*Phvul.002G121800.1*	pvu_Phvul.002G121800	606	95.2%
110	*Physcomitrella patens*	ppa	*Pp3c16_8950V3.1*	ppa_Pp3c16_8950	605	93.9%
111	*Physcomitrella patens*	ppa	*Pp3c25_1990V3.1*	ppa_Pp3c25_1990	605	93.6%
112	*Populus trichocarpa*	ptr	*Potri.003G045700.1*	ptr_Potri.003G045700	611	94.7%
113	*Populus trichocarpa*	ptr	*Potri.004G235900.1*	ptr_Potri.004G235900	605	95.5%
114	*Prunus persica*	ppe	*Prupe.1G366500.1*	ppe_Prupe.1G366500	605	95.0%
115	*Prunus persica*	ppe	*Prupe.3G017900.1*	ppe_Prupe.3G017900	601	87.4%
116	*Prunus persica*	ppe	*Prupe.3G018600.1*	ppe_Prupe.3G018600	605	94.9%
117	*Ricinus communis*	rco	*29756.m000511*	rco_29756.m000511	591	95.4%
118	*Salix purpurea*	spu	*SapurV1A.1342s0120.1*	spu_SapurV1A.1342s0120	605	95.4%
119	*Salix purpurea*	spu	*SapurV1A.2493s0010.1*	spu_SapurV1A.2493s0010	605	95.4%
120	*Setaria italica*	sit	*Seita.1G128500.1*	sit_Seita.1G128500	604	79.1%
121	*Setaria italica*	sit	*Seita.8G145600.1*	sit_Seita.8G145600	604	95.4%
122	*Setaria viridis*	svi	*Sevir.1G127100.1*	svi_Sevir.1G127100	604	79.1%
123	*Setaria viridis*	svi	*Sevir.8G155600.1*	svi_Sevir.8G155600	604	95.4%
124	*Solanum lycopersicum*	sly	*Solyc08g075360.1.1*	sly_Solyc08g075360.1	606	93.4%
125	*Solanum tuberosum*	stu	*PGSC0003DMT400051207*	stu_PGSC0003DMG400019889	605	95.5%
126	*Sorghum bicolor*	sbi	*Sobic.001G447300.1*	sbi_Sobic.001G447300	604	95.5%
127	*Sorghum bicolor*	sbi	*Sobic.004G128100.1*	sbi_Sobic.004G128100	606	78.1%
128	*Sphagnum fallax*	sfa	*Sphfalx0014s0125.1*	sfa_Sphfalx0014s0125	605	93.6%
129	*Sphagnum fallax*	sfa	*Sphfalx0028s0121.1*	sfa_Sphfalx0028s0121	605	94.2%
130	*Spirodela polyrhiza*	spo	*Spipo14G0028600*	spo_Spipo14G0028600	605	94.7%
131	*Zea mays Ensembl-18*	zmays	*GRMZM2G006178_T01*	zmays_GRMZM2G006178	604	95.4%
132	*Zea mays Ensembl-18*	zmays	*GRMZM2G109121_T02*	zmays_GRMZM2G109121	604	95.2%
133	*Zostera marina*	zmarina	*Zosma105g00220.1*	zmarina_Zosma105g00220	605	92.4%
134	*Theobroma cacao*	tca	*Thecc1EG016698t1*	tca_Thecc1EG016698	605	95.5%
135	*Trifolium pratense*	tpr	*Tp57577_TGAC_v2_mRNA6813*	tpr_gene6580_mRNA6813	603	89.0%
136	*Trifolium pratense*	tpr	*Tp57577_TGAC_v2_mRNA9295*	tpr_gene8993_mRNA9295	606	94.0%
137	*Triticum aestivum*	tae	*TraesCS1A02G218400.2*	tae_TraesCS1A02G218400.2	599	87.6%
138	*Triticum aestivum*	tae	*TraesCS1B02G231900.1*	tae_TraesCS1B02G231900.1	599	87.8%
139	*Triticum aestivum*	tae	*TraesCS1D02G220000.1*	tae_TraesCS1D02G220000.1	599	87.6%
140	*Triticum aestivum*	tae	*TraesCS2B02G600300.1*	tae_TraesCS2B02G600300.1	604	92.5%

No	Latin species name	Species acronym in this study	Locus/gene/transcript name	Name used within this study	Peptide length	Amino acid similarity with AtABCE2
141	*Triticum aestivum*	tae	*TraesCS2D02G594500.1*	tae_TraesCS2D02G594500.1	604	92.4%
142	*Triticum aestivum*	tae	*TraesCS4A02G143000.4*	tae_TraesCS4A02G143000.4	604	93.5%
143	*Triticum aestivum*	tae	*TraesCS4B02G160000.1*	tae_TraesCS4B02G160000.1	604	93.5%
144	*Triticum aestivum*	tae	*TraesCSU02G036500.1*	tae_TraesCSU02G036500.1	604	92.2%
145	*Triticum dicoccoides*	tdi	*TRIDC1AG033010.1*	tdi_TRIDC1AG033010.1	596	87.8%
146	*Triticum dicoccoides*	tdi	*TRIDC1BG038390.1*	tdi_TRIDC1BG038390.1	602	86.9%
147	*Triticum dicoccoides*	tdi	*TRIDC4AG020920.1*	tdi_TRIDC4AG020920.1	604	93.5%
148	*Triticum dicoccoides*	tdi	*TRIDC4BG026550.1*	tdi_TRIDC4BG026550.1	604	93.5%
149	*Vigna angularis*	van	*LR48_Vigan06g066400; KOM45358*	van_LR48_Vigan06g066400:KOM45358	606	95.0%
150	*Vigna radiata*	vra	*Vradi11g07590.1*	vra_Vradi11g07590.1	606	95.0%
151	*Vitis vinifera*	vvi	*GSVIVT01036876001*	vvi_GSVIVG01036876001	625	95.9%
152	*Volvox carteri*	vca	*Vocar.0036s0015.1*	vca_Vocar.0036s0015	619	86.9%
