# Supplementary figures and images for: Phylogenetic insight into ABCE gene subfamily in plants
Source: Front Genet. 2024 Jun 7;15:1408665. doi: 10.3389/fgene.2024.1408665 (PMC11190730; doi:10.3389/fgene.2024.1408665)

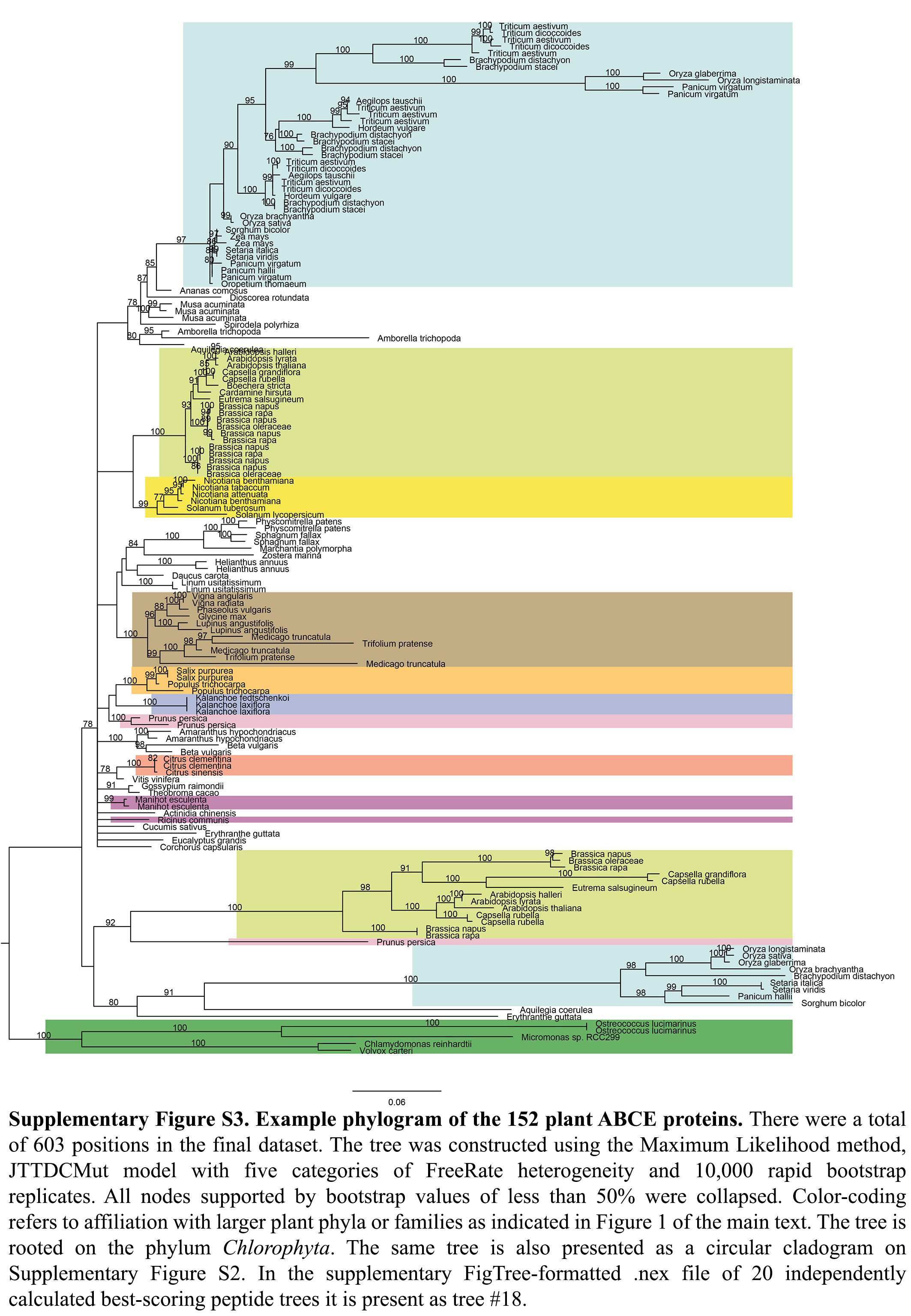

Supplement: Supplementary file 3 [file Image3.jpg]

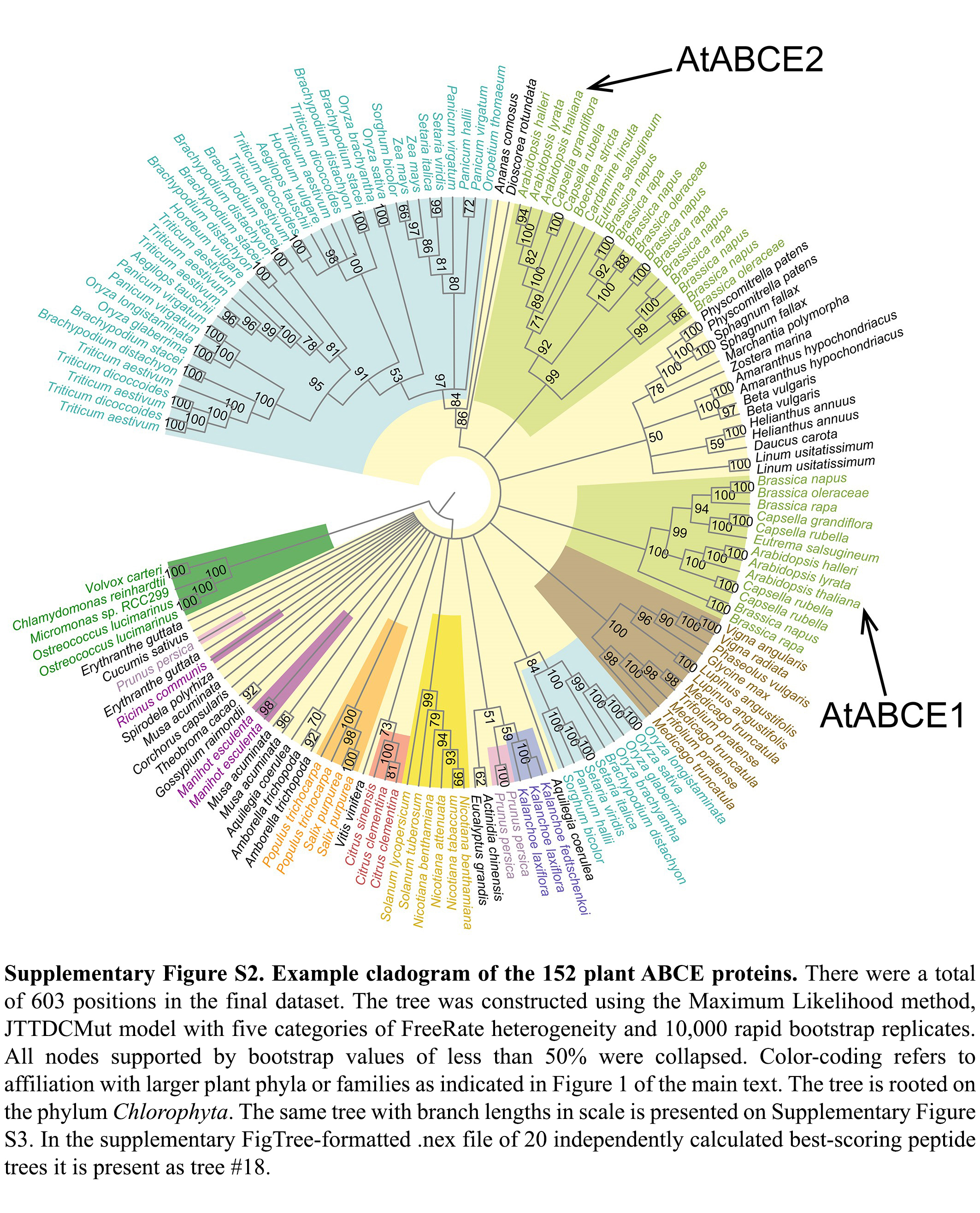

Supplement: Supplementary file 4 [file Image2.jpg]

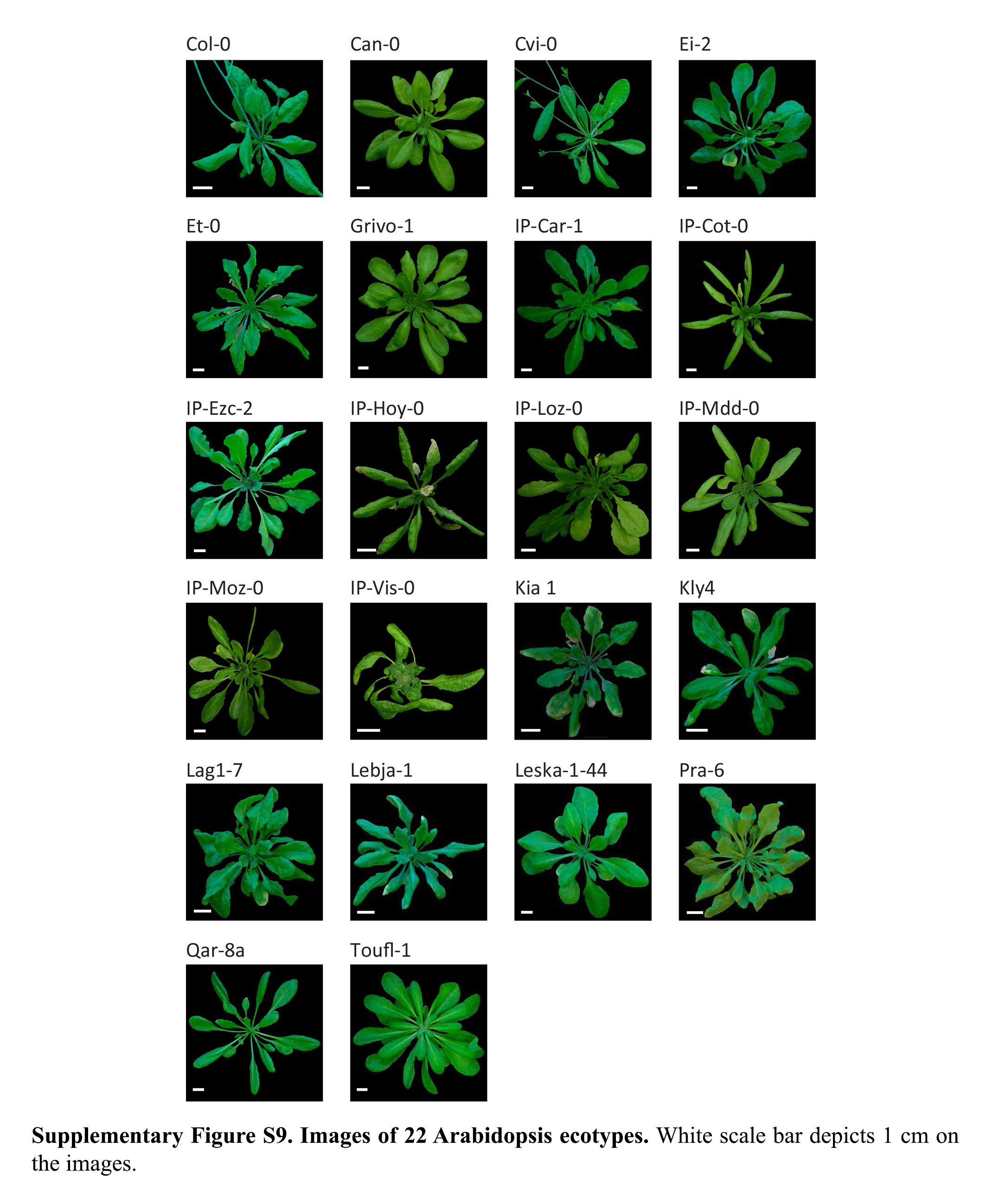

Supplement: Supplementary file 5 [file Image9.JPEG]

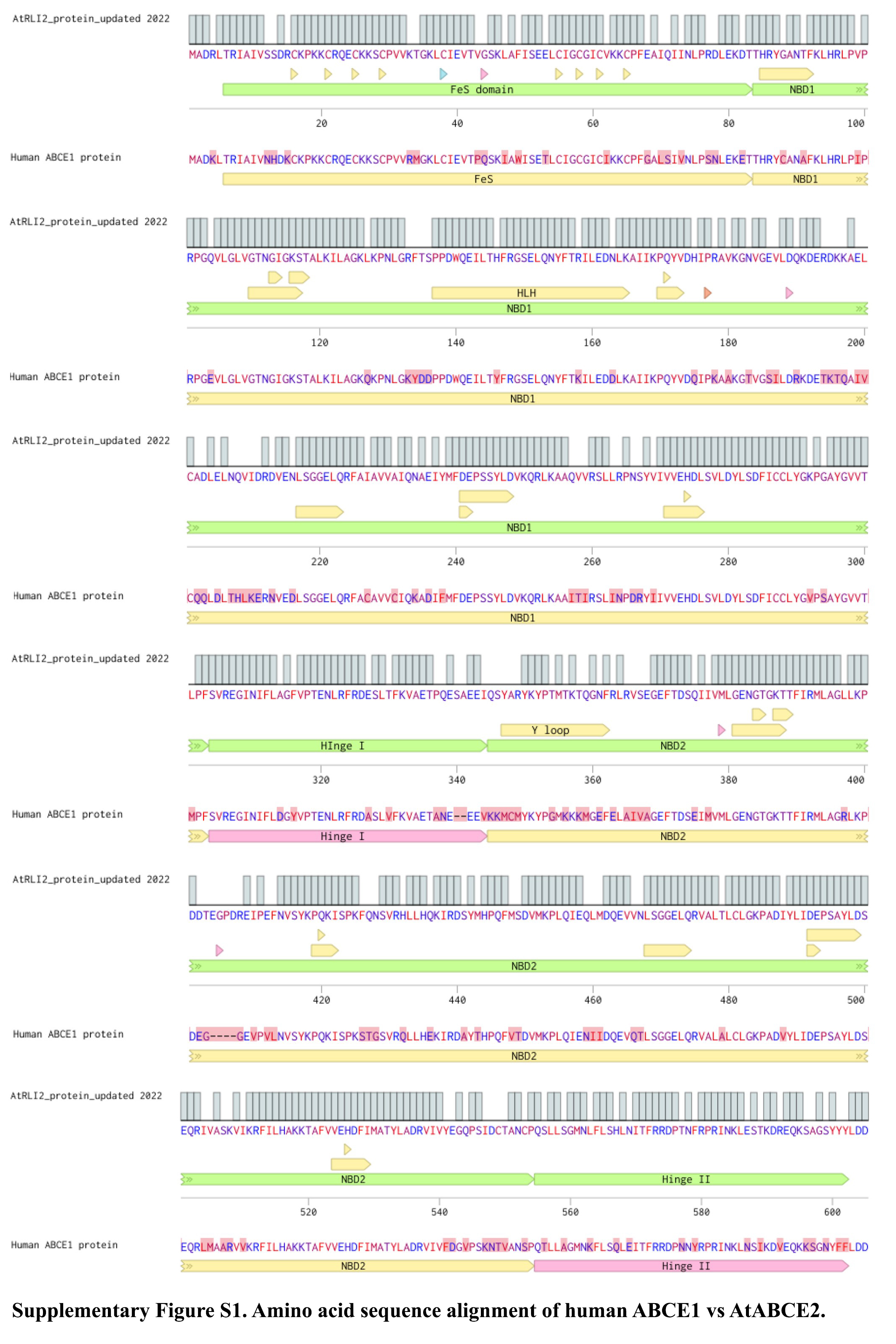

Supplement: Supplementary file 6 [file Image1.JPEG]

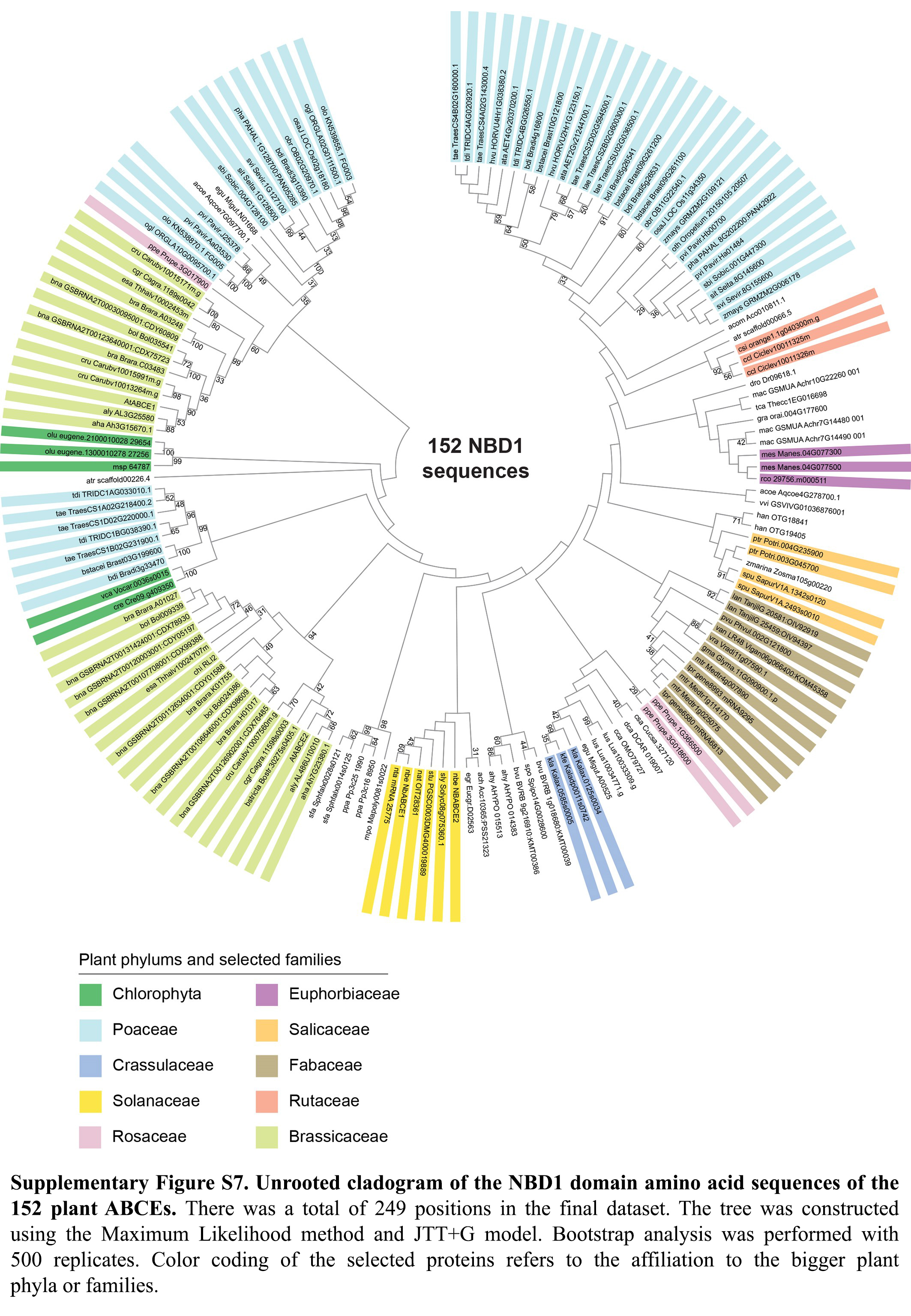

Supplement: Supplementary file 7 [file Image7.JPEG]

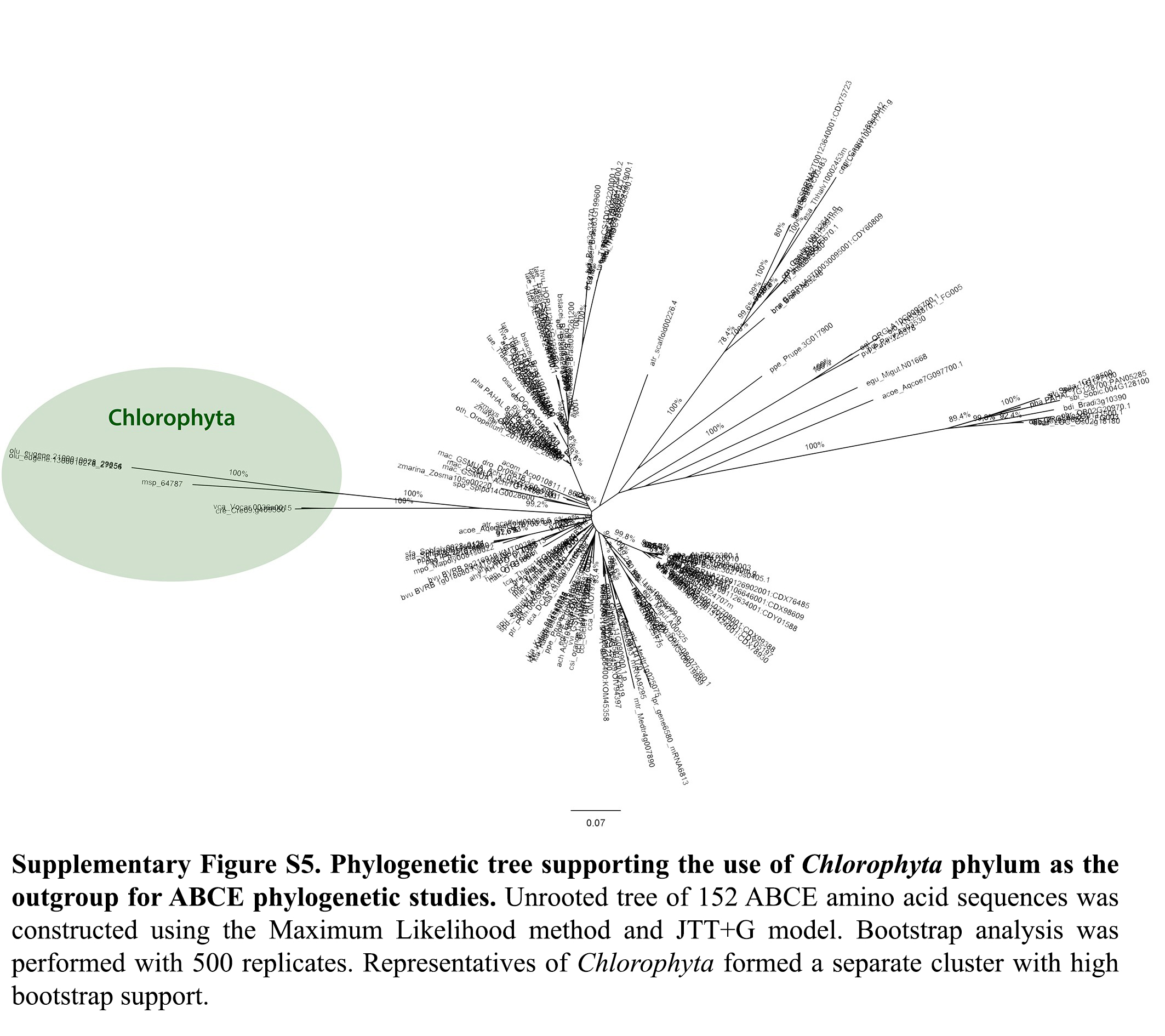

Supplement: Supplementary file 8 [file Image5.JPEG]

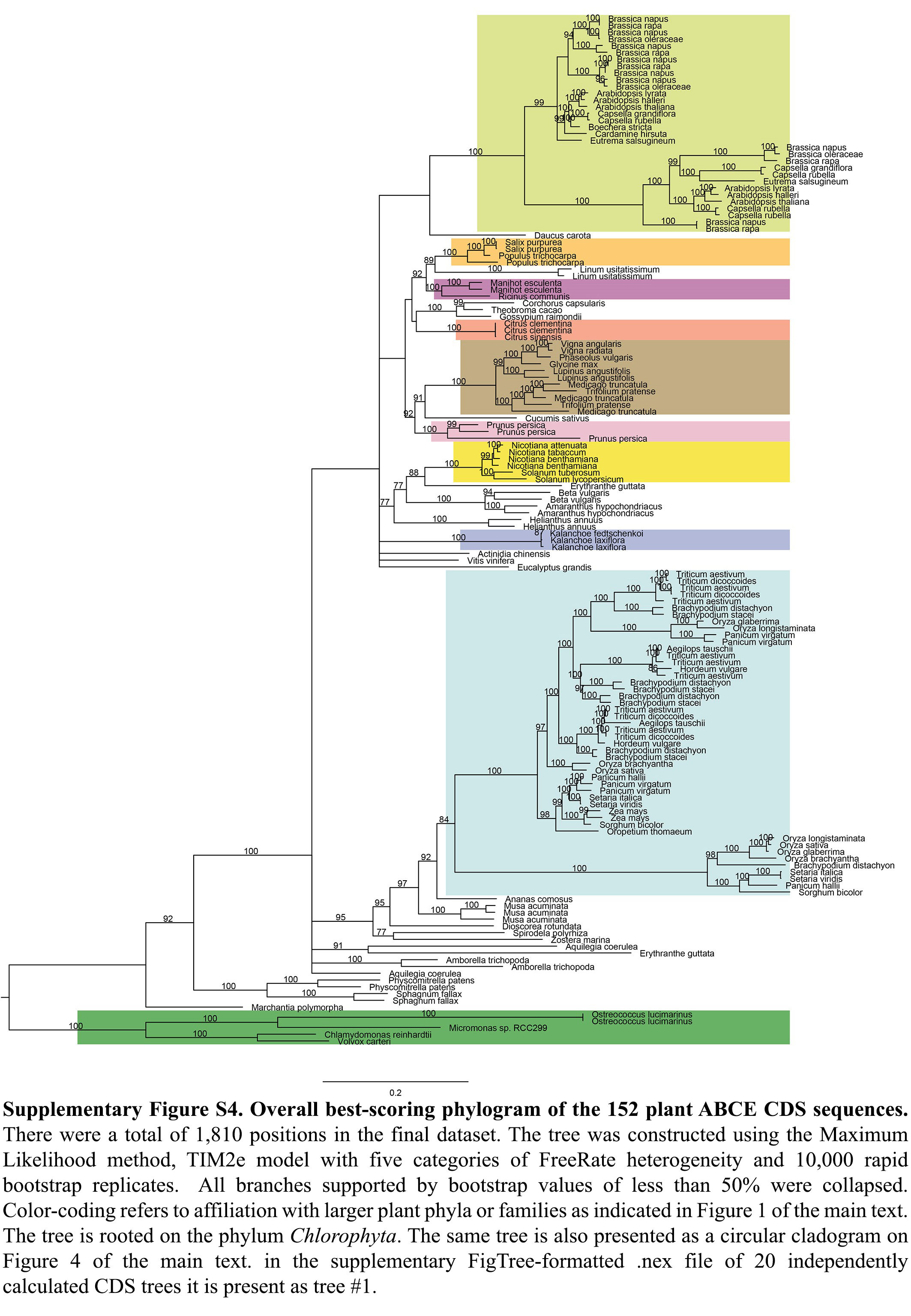

Supplement: Supplementary file 12 [file Image4.jpg]

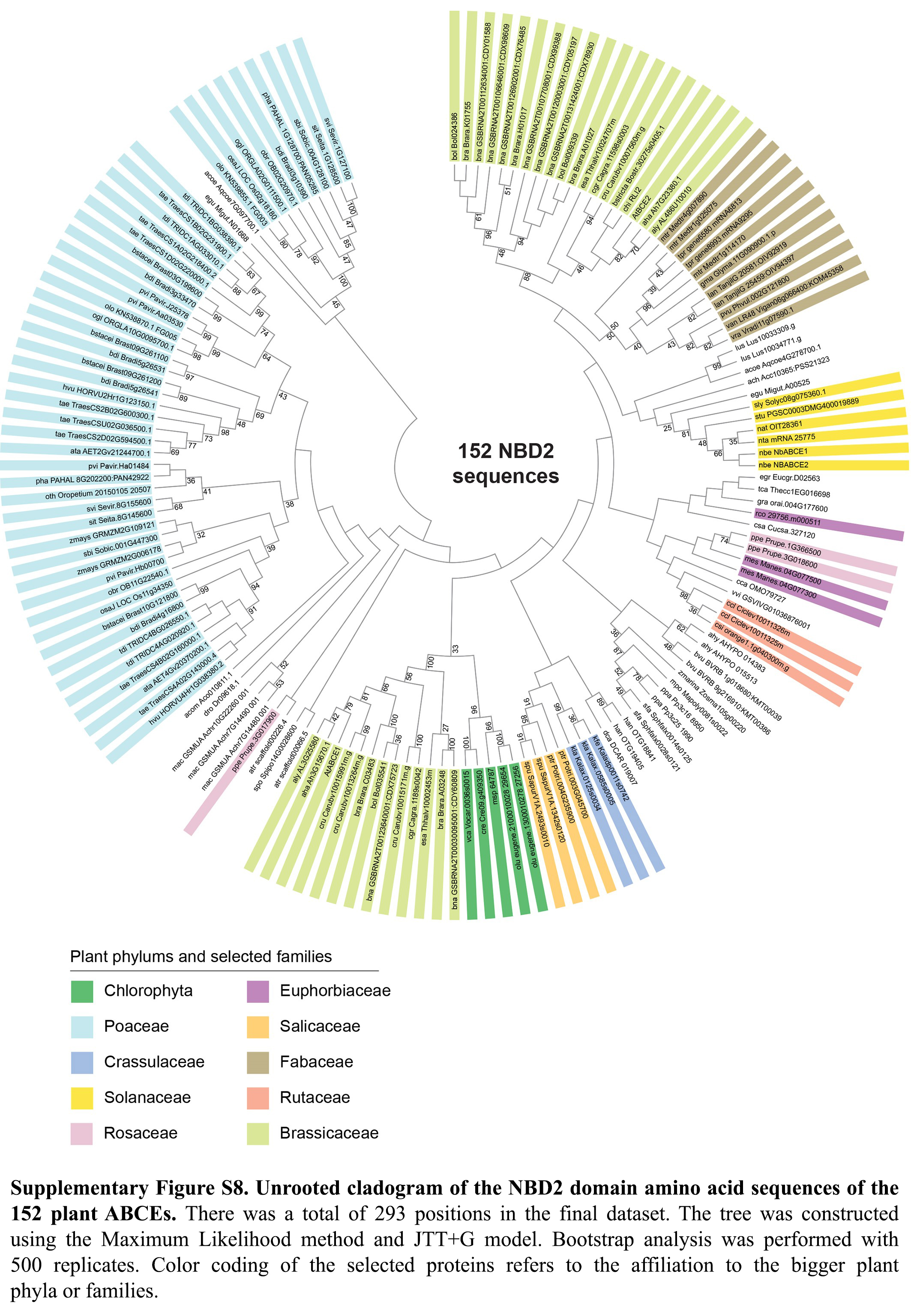

Supplement: Supplementary file 13 [file Image8.JPEG]

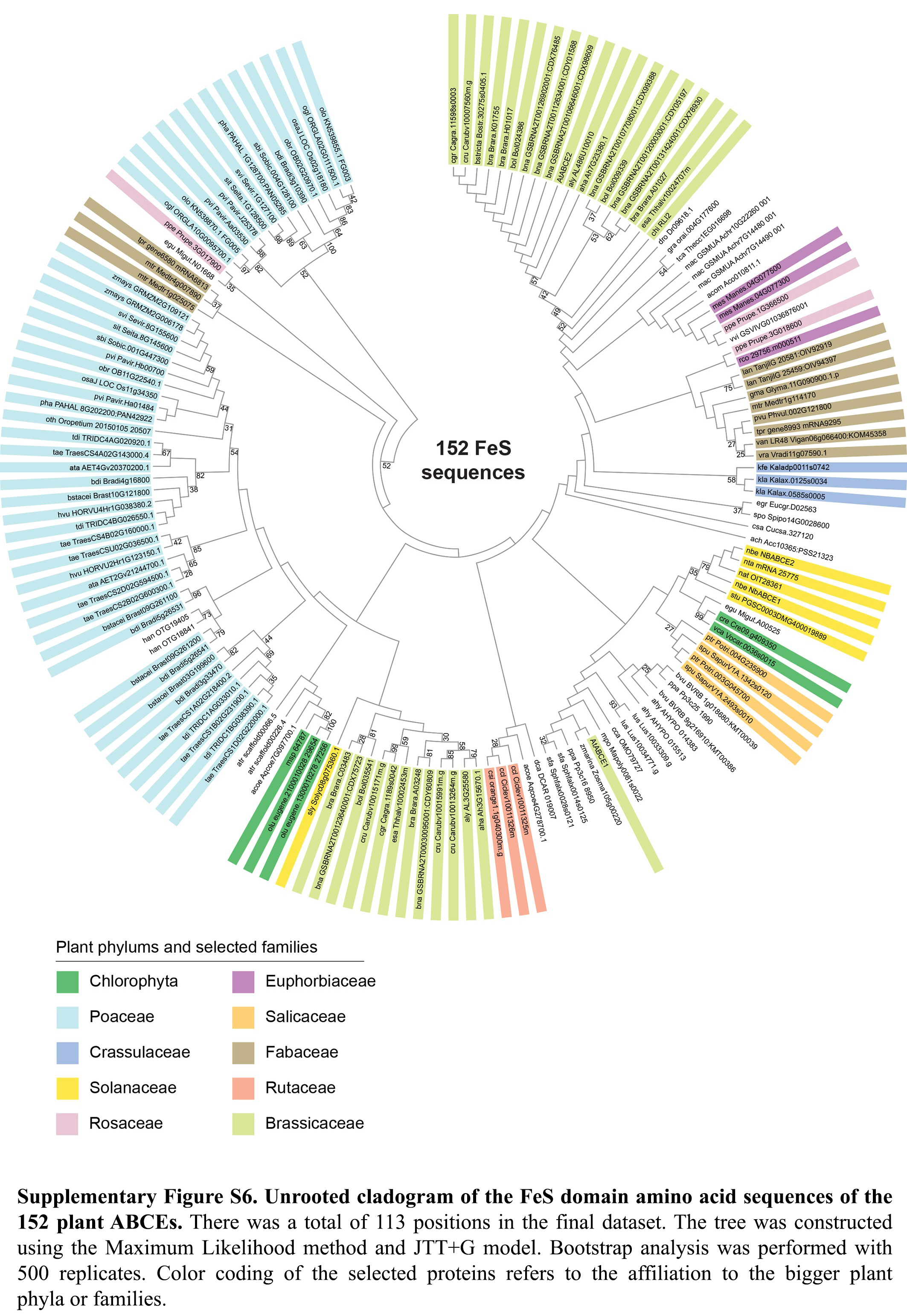

Supplement: Supplementary file 14 [file Image6.JPEG]
