# Supplementary material for: Phylogenetic insight into ABCE gene subfamily in plants
Source: Front Genet. 2024 Jun 7;15:1408665. doi: 10.3389/fgene.2024.1408665 (PMC11190730; doi:10.3389/fgene.2024.1408665)
Supplement: Supplementary file 9 [file DataSheet3.PDF]

Supplementary Table S3. Whole-genome duplications (WGDs) detected in the plant species analyzed in the study. Data is based on the results of One Thousand Plant Transcriptomes Initiative published in 2019.

Species	No of *ABCE* genes	No of WGDs	WGDs
Volvox carteri	1	0	
Ostreococcus lucimarinus	2	0	
Micromonas sp. RCC299	1	0	
Marchantia polymorpha	1	0	
Chlamydomonas reinhardtii	1	1	CHMOα
Physcomitrella patens	2	1	PHPAα
Sphagnum fallax	2	1	SPPAα
Amborella trichopoda	2	2	GINKα, AMBOα
Zostera marina	1	2	GINKα, AMBOα
Brachypodium stacei	4	2	GINKα, AMBOα
Brachypodium distachyon	5	2	GINKα, AMBOα
Hordeum vulgare	2	2	GINKα, AMBOα
Aegilops tauschii	2	2	GINKα, AMBOα
Triticum dicoccoides	4	2	GINKα, AMBOα
Triticum aestivum	8	2	GINKα, AMBOα
Beta vulgaris	2	3	GINKα, AMBOα, ARTHγ
Vitis vinifera	1	3	GINKα, AMBOα, ARTHγ
Prunus persica	3	3	GINKα, AMBOα, ARTHγ
Ricinus communis	1	3	GINKα, AMBOα, ARTHγ
Citrus sinensis	1	3	GINKα, AMBOα, ARTHγ
Citrus clementina	2	3	GINKα, AMBOα, ARTHγ
Theobroma cacao	1	3	GINKα, AMBOα, ARTHγ
Helianthus annuus	2	3	GINKα, AMBOα, ARTHγ
Daucus carota	1	3	GINKα, AMBOα, ARTHγ
Cucumis sativus	1	3	GINKα, AMBOα, ARTHγ
Medicago truncatula	3	3	GINKα, AMBOα, ARTHγ
Trifolium pratense	2	3	GINKα, AMBOα, ARTHγ
Vigna radiata	1	3	GINKα, AMBOα, ARTHγ
Vigna angularis	1	3	GINKα, AMBOα, ARTHγ
Gossypium raimondii	1	3	GINKα, AMBOα, ARTHγ
Corchorus capsularis	1	3	GINKα, AMBOα, ARTHγ
Cardamine hirsuta	1	3	GINKα, AMBOα, ARTHγ
Boechera stricta	1	3	GINKα, AMBOα, ARTHγ
Eutrema salsugineum	2	3	GINKα, AMBOα, ARTHγ
Ananas comosus	1	3	GINKα, AMBOα, ORSAγ
Oropetium thomaeum	1	3	GINKα, AMBOα, ORSAγ
Aquilegia coerulea	2	3	GINKα, AMBOα, PASOβ
Spirodela polyrhiza	1	3	GINKα, AMBOα, SPPOα
Amaranthus hypochondriacus	2	4	GINKα, AMBOα, ARTHγ, AMREα
Erythranthe guttata	2	4	GINKα, AMBOα, ARTHγ, ANMAα
Kalanchoe fedtschenkoi	1	4	GINKα, AMBOα, ARTHγ, CRAPα
Kalanchoe laxiflora	2	4	GINKα, AMBOα, ARTHγ, CRAPα
Phaseolus vulgaris	1	4	GINKα, AMBOα, ARTHγ, GLSOβ
Linum usitatissimum	2	4	GINKα, AMBOα, ARTHγ, LIUSα
Solanum lycopersicum	1	4	GINKα, AMBOα, ARTHγ, LYBAα
Solanum tuberosum	1	4	GINKα, AMBOα, ARTHγ, LYBAα
Nicotiana tabacum	1	4	GINKα, AMBOα, ARTHγ, LYBAα
Nicotiana attenuata	1	4	GINKα, AMBOα, ARTHγ, LYBAα

**Species**	**No of *ABCE* genes**	**No of WGDs**	**WGDs**
Nicotiana benthamiana	2	4	GINKα, AMBOα, ARTHγ, LYBAα
Manihot esculenta	2	4	GINKα, AMBOα, ARTHγ, MAESα
Populus trichocarpa	2	4	GINKα, AMBOα, ARTHγ, SAACα
Salix purpurea	2	4	GINKα, AMBOα, ARTHγ, SAACα
Eucalyptus grandis	1	4	GINKα, AMBOα, ARTHγ, SYMIα
Dioscorea rotundata	1	4	GINKα, AMBOα, ORSAγ, DIVIα
Actinidia chinensis Red5	1	5	GINKα, AMBOα, ARTHγ, ACCHα, ACCHβ
Glycine max	1	5	GINKα, AMBOα, ARTHγ, GLSOα, GLSOβ
Lupinus angustifolius	2	5	GINKα, AMBOα, ARTHγ, LUPOα, GLSOβ
Musa acuminata	3	5	GINKα, AMBOα, ORSAγ, MUACα, MALEα
Sorghum bicolor	2	5	GINKα, AMBOα, ORSAγ, ORSAα, ORSAβ
Panicum virgatum	4	5	GINKα, AMBOα, ORSAγ, ORSAα, ORSAβ
Panicum hallii ecotype FIL2	2	5	GINKα, AMBOα, ORSAγ, ORSAα, ORSAβ
Oryza glaberrima	2	5	GINKα, AMBOα, ORSAγ, ORSAα, ORSAβ
Oryza longistaminata	2	5	GINKα, AMBOα, ORSAγ, ORSAα, ORSAβ
Oryza brachyantha	2	5	GINKα, AMBOα, ORSAγ, ORSAα, ORSAβ
Oryza sativa Japonica Group	2	5	GINKα, AMBOα, ORSAγ, ORSAα, ORSAβ
Zea mays	2	5	GINKα, AMBOα, ORSAγ, ORSAα, ORSAβ
Setaria italica	2	5	GINKα, AMBOα, ORSAγ, ORSAα, ORSAβ
Setaria viridis	2	5	GINKα, AMBOα, ORSAγ, ORSAα, ORSAβ
Brassica napus	8	6	GINKα, AMBOα, ARTHγ, BRNIα, ARTHα, ARTHβ
Brassica oleracea	3	6	GINKα, AMBOα, ARTHγ, BRNIα, ARTHα, ARTHβ
Brassica rapa	5	6	GINKα, AMBOα, ARTHγ, BRNIα, ARTHα, ARTHβ
Capsella grandiflora	2	6	GINKα, AMBOα, ARTHγ, BRNIα, ARTHα, ARTHβ
Capsella rubella	4	6	GINKα, AMBOα, ARTHγ, BRNIα, ARTHα, ARTHβ
Arabidopsis thaliana	2	6	GINKα, AMBOα, ARTHγ, BRNIα, ARTHα, ARTHβ
Arabidopsis lyrata	2	6	GINKα, AMBOα, ARTHγ, BRNIα, ARTHα, ARTHβ
Arabidopsis halleri	2	6	GINKα, AMBOα, ARTHγ, BRNIα, ARTHα, ARTHβ
