# Supplementary material for: Phylogenetic insight into ABCE gene subfamily in plants
Source: Front Genet. 2024 Jun 7;15:1408665. doi: 10.3389/fgene.2024.1408665 (PMC11190730; doi:10.3389/fgene.2024.1408665)
Supplement: Supplementary file 10 [file DataSheet1.PDF]

Table S1. Primers used within this study.

Primer name	Primer sequence	Comments
**RLI1_F4**	5’ - GAAGGAATCAACGTGTTCTTGG - 3’	For sequencing *AtABCE1* SNPs
**RLI1_R4**	5’ - CCATCTCTCTCTGACCGATC - 3’	For sequencing *AtABCE1* SNPs
**RLI1_F3**	5’ - TTGCAGGCTTCCAATTCCAC - 3’	For sequencing *AtABCE1* SNPs
**RLI1_R3**	5’ - CTGAAGGTCAGGGATTCATCC - 3’	For sequencing *AtABCE1* SNPs
**RLI1_s**	5’ - TTGACTTGTGTATCTTGTAT - 3’	For sequencing *AtABCE1* SNPs
**RLI1_R2**	5’ - GCTGGAAAACTCAAACCAAATC - 3’	For sequencing *AtABCE1* SNPs
**RLI2_F3**	5’ - CAGAGTCCTCCAGACTGGCAAG - 3’	For sequencing *AtABCE2* SNPs
**RLI2_R3**	5’ - CCTTGAAGGTCAAAGATTCATCTC - 3’	For sequencing *AtABCE2* SNPs
**RLI2_RP**	5’ - CTGTAGGAACAAATCCAGCCA - 3’	For sequencing *AtABCE2* SNPs
**RLI2_LP**	5’ - TTCTTGGTCTGAAATTGGTGG - 3’	For sequencing *AtABCE2* SNPs
**RLI2_F1**	5’ - TGTTATTTCGTTCCTTTGCCTT - 3’	For sequencing *AtABCE2* SNPs
**RLI2_as**	5’ - CATCTTGGATATCGGAAAGAGC - 3’	For sequencing *AtABCE2* SNPs
