# Supplementary material for: Phylogenetic insight into ABCE gene subfamily in plants
Source: Front Genet. 2024 Jun 7;15:1408665. doi: 10.3389/fgene.2024.1408665 (PMC11190730; doi:10.3389/fgene.2024.1408665)
Supplement: Supplementary file 11 [file DataSheet5.PDF]

Supplementary Table S5. Non-synonymous SNPs found in *AtABCE1* and *AtABCE2* .

AtABCE2

position	SNP	Codon change	Ecotype	Selected ecotypes	Comment
10502255	G44S/Gly44Ser/c.130G>A	Ggt/Agt	54 ecotypes: PYL-6, Brösarp-15-138, Ängsö-59-422, Mc-1, App1-14, App1-16, Fly2-1, Fly2-2, Hov1-7, Hov3-5, Kni-1, Rev-3, Stu-2, T1000, T1010, T1160, T610, T670, T800, T860, T930, TDr-2, TDr-8, Tomegap-2, Ei-2, Ull2-5, Edi-0, Gie-0, Kl-5, Kb-0, Nw-0, Oy-0, Petergof, Rome-1, Su-0, Nc-1, Algutsrum, Gul1-2, Hov4-1, Lund, Rev-1, St-0, FlyA 3, Kia 1, Ull-A-1, IP-Jim-1, IP-Moc-11, IP-Vaz-0, IP-Gud-3, IP-Mac-0, IP-Rib-1, IP-Urd-1, IP-Vas-0, Pra-6	**Ei-2, Kia 1, Pra-6**	not conserved
10503064	D189E/Asp189Glu/c.567C>A	gaC/gaA	Ost-0	Ost-0: not available	
10503813	M379T/Met379Thr/c.1136T>C	aTg/aCg	IP-Car-1	**IP-Car-1**	not conserved
10503980	G405A/Gly405Ala/c.1214G>C	gGa/gCa	Can-0	**Can-0**	not conserved

AtABCE1

position	SNP	Codon change	Ecotypes	Selected ecotypes	Comment
4458801	p.Lys588*/c.1762A>T	Aag/Tag	Kly-4	**Kly-4**	stop gain mutation
4458848	p.Arg572Leu/c.1715G>T	cGg/cTg	2 ecotypes: Aitba-1, Toufl-1	**Toufl-1**	conserved Arg in Hinge II subdomain
4458862	p.Asn567Lys/c.1701C>A	aaC/aaA	IP-Moc-11	IP-Moc-11: not available	
4458966	p.His561Leu/c.1682A>T	cAc/cTc	997 ecotypes	all 21 ecotypes	not conserved
4459002	p.Ala549Gly/c.1646C>G	gCt/gGt	6 ecotypes: Lag1-2, Lag1-5, Lag1-6, Lag1-7, Lag2-4, Qar-8a	**Qar-8a, Lag1-7**	substitution for similar AA residue
4459219	p.Thr477Ala/c.1429A>G	Act/Gct	Uod-7		not conserved
4459291	p.Leu453Phe/c.1357C>T	Ctt/Ttt	7 ecotypes: IP-Cem-0, IP-Cor-0, IP-Fun-0, IP-Hum-2, IP-Nac-0, IP-Pun-0, IP-Ven-0		substitution for similar AA residue
4459327	p.Ala441Thr/c.1321G>A	Gca/Aca	28 ecotypes: Hovdala-2, Hovdala-2, Cvi-0, Kz-9, Ak-1, Kn-0, Sei-0, Zu-1, Lag1-2, Lag1-5, Lag1-6, Lag1-7, Lag2-4, Lag2-7, Lag2-10, Bak-5, EkS 2, EkS 3, Kolyv-3, Goced-1, Podvi-1, Stara-1, Leska-1-44, Koren-1, Malak-1, Epidauros-1, Qar-8a, IP-Mdd-0, Bak-2	**IP-Mdd-0, Cvi-0, Leska-1-44, Qar-8a, Lag1-7**	not conserved
4459351	p.His433Asp/c.1297C>G	Cat/Gat	IP-Cat-0		not conserved
4459411	p.Val413Leu/c.1237G>C	Gtg/Ctg	RUM-20		substitution for similar AA residue
4459438	p.Val404Ile/c.1210G>A	Gta/Ata	Zdarec3		substitution for similar AA residue
4459443	p.Glu402Val/c.1205A>T	gAg/gTg	Faneronemi-3		not conserved
4459450	p.Arg400Ser/c.1198C>A	Cgt/Agt	2 ecotypes: Kz-9, Kolyv-3		not conserved
4459450	p.Arg400Cys/c.1198C>T	Cgt/Tgt	IP-Ses-0		not conserved
4459453	p.Pro399Thr/c.1195C>A	Cca/Aca	Grivo-1	**Grivo-1**	substitution of conserved Pro in NBD2
4459607	p.Asp373His/c.1117G>C	Gac/Cac	6 ecotypes: Lag1-2, Lag1-5, Lag1-6, Lag1-7, Lag2-4, Qar-8a	**Lag1-7, Qar-8a**	substitution of conserved Asp in NBD2
4459652	p.Gln358Lys/c.1072C>A	Caa/Aaa	IP-Ldd-0, Furni-1, Iasi-1, Bolin-1		not conserved
4459687	p.Ser346Phe/c.1037C>T	tCc/tTc	App1-14		not conserved
4459714	p.Thr337Arg/c.1010C>G	aCa/aGa	21 ecotypes: Leb-3, Karag-2, Basta-1, Basta-2, Basta-3, Chaba-2, Lebja-1, Lebja-2, Masl-1, Nosov-1, Noveg-1, Noveg-2, Noveg-3, Panke-1, Rakit-1, Rakit-2, Rakit-3, Sever-1, Stepn-2, Stepn-1, Kidr-1	**Lebja-1**	not conserved
4459823	p.Ser328Phe/c.983C>T	tCc/tTc	Cimin-1		not conserved
4459839	p.Arg323Cys/c.967C>T	Cgt/Tgt	IP-Moz-0	**IP-Moz-0**	conserved Arg in Hinge I subdomain
4459928	p.Pro293Gln/c.878C>A	cCa/cAa	IP-Vis-0	**IP-Vis-0**	substitution of conserved Pro in NBD1
4459995	p.Val271Ile/c.811G>A	Gtt/Att	Cvi-0	**Cvi-0**	substitution for similar AA residue
4460286	p.Leu206Phe/c.618G>T	ttG/ttT	App1-12		substitution of conserved Leu in NBD1
4460360	p.Gly182Ser/c.544G>A	Ggt/Agt	8 ecotypes: Jl-3, IP-All-0, IP-Vin-0, IP-Bra-0, IP-Cot-0, IP-Vas-0, Lecho-1, Dobra-1	**IP-Cot-0**	substitution of conserved Gly in NBD1

position	SNP	Codon change	Ecotype	Selected ecotypes	Comment
4460508	p.Val160Ile/c.478G>A	Gta/Ata	[illegible] App1-16, Boo2-3, Fly2-1, Fly2-2, Kni-1, T1010, T1160, T610, T670, T710, T780, T800, T850, T880, T900, T930, T960, T970, T980, Ga-0, LL-0, Ts-5, Wt-5, Et-0, Fr-2, Hs-0, Mnz-0, Ob-0, Old-1, Or-0, Rsch-4, Sf-1, Kent, Bå1-2, Bla-1, Lund, FlyA 3, HolA-1 2, Ull-A-1, IP-Ang-0, IP-Coc-1, IP-Elb-0, IP-Gua-1, IP-Hor-0, IP-Moc-11, IP-Mon-5, IP-Rds-0, IP-Tam-0, IP-Aru-0, IP-Cas-0, IP-Mac-0, IP-Mie-1, IP-Sal-0, IP-Tri-0, ESP-1-11, ARGE-1-15, Qui-0	**Et-0**	substitution for similar AA residue
4460511	p.Val159Leu/c.475G>C	Gta/Cta	UKSE06-639		substitution for similar AA residue
4460513	p.Arg158Gln/c.473G>A	cGa/cAa	Et-0	**Et-0**	not conserved
4460571	p.Asp139Asn/c.415G>A	Gac/Aac	IP-Mdd-0	**IP-Mdd-0**	not conserved
4460697	p.Pro129Gln/c.386C>A	cCa/cAa	15 ecotypes: Ak-1, Kn-0, Sei-0, Zu-1, Lag1-2, Lag1-5, Lag1-6, Lag1-7, Lag2-4, Podvi-1, Stara-1, Leska-1-44, Koren-1, Qar-8a, IP-Mdd-0	**Lag1-7, Qar-8a, IP-Mdd-0, Leska1-44**	substitution of conserved Pro in NBD1
4460709	p.Gly125Glu/c.374G>A	gGa/gAa	35 ecotypes: Dör-10, Dra-3, Eden-1, Eden-5, Eden-6, Eden-7, Grön-5, Nyl-2, Nyl-7, TÄL 03, TEDEN 02, TFÄ 04, TFÄ 06, TFÄ 07, TFÄ 08, TGR 01, TNY 04, Fäb-2, Fäb-4, Tamm-2, Tamm-27, Sanna-2, FäL 1, Grön 12, Nyl 13, IP-Hoy-0, IP-Ria-0, IP-Are-0, IP-Boa-0, IP-Coy-0, IP-Elp-0, IP-Leg-0, IP-Loz-0, IP-Pad-0, IP-Pva-1	**IP-Hoy-0, IP-Loz-0**	substitution of conserved Gly in NBD1
4460719	p.Ile122Leu/c.364A>C	Atc/Ctc	IP-Adc-5		substitution for similar AA residue
4460908	p.Arg86Gln/c.257G>A	cGa/cAa	19 ecotypes: Draha2, DraII-6, DraIV 1-8, DraIV 2-9, DraIV 6-22, Duk, UduI 4-9, ZdrI 2-21, Dra-0, Jm-0, Kyoto, Petergof, Da(1)-12, DraIII-1, Rak-2, IP-Bos-0, IP-Ezc-2, Galdo-1, Monte-1	**IP-Ezc-2**	substitution of conserved Arg in Y-loop I
4460921	p.Asp82Tyr/c.244G>T	Gat/Tat	Spro 1		not conserved
4461106	p.Ile50Leu/c.148A>C	Ata/Cta	2 ecotypes: Sr:3, Strand-1		substitution for similar AA residue
